# Aging and Intermittent Fasting Impact on Transcriptional Regulation and Physiological Responses of Adult *Drosophila* Neuronal and Muscle Tissues

**DOI:** 10.3390/ijms19041140

**Published:** 2018-04-10

**Authors:** Sharon Zhang, Eric P. Ratliff, Brandon Molina, Nadja El-Mecharrafie, Jessica Mastroianni, Roxanne W. Kotzebue, Madhulika Achal, Ruth E. Mauntz, Arysa Gonzalez, Ayeh Barekat, William A. Bray, Andrew M. Macias, Daniel Daugherty, Greg L. Harris, Robert A. Edwards, Kim D. Finley

**Affiliations:** 1 Donald P. Shiley BioScience Center, San Diego State University, San Diego, CA 92182, USA; sharonzh10@yahoo.com (S.Z.); epratliff@hotmail.com (E.P.R.); Brandonrm.16@gmail.com (B.M.); nadjaelm@outlook.com (N.E.-M.); jmfmastroianni@yahoo.com (J.M.); rkotzebue@gmail.com (R.W.K.); madhulika.achal@gmail.com (M.A.); rmauntz@gmail.com (R.E.M.); gonzalez.arysa@gmail.com (A.G.); ayeh.sdsu@gmail.com (A.B.); yossarianassyrian@gmail.com (W.A.B.); maciasandrew01@gmail.com (A.M.M.); 2 Biological and Medical Informatics Research Center, San Diego State University, San Diego, CA 92182, USA; redwards@sdsu.edu; 3 Department of Biology, San Diego State University, San Diego, CA 92182, USA; gharris@sdsu.edu; 4 Sanford Consortium for Regenerative Medicine, 2880 Torrey Pines Scenic Dr., La Jolla, CA 92037, USA; 5 The Salk Institute for Biological Studies, Cellular Neurobiology, 10010 North Torrey Pines Road, La Jolla, CA 92037, USA; ddaugherty@salk.edu

**Keywords:** aging, aging-delaying interventions, metabolism, cellular proteostasis, neural degeneration, intermittent fasting, RNA-sequencing, *Drosophila*

## Abstract

The progressive decline of the nervous system, including protein aggregate formation, reflects the subtle dysregulation of multiple functional pathways. Our previous work has shown intermittent fasting (IF) enhances longevity, maintains adult behaviors and reduces aggregates, in part, by promoting autophagic function in the aging *Drosophila* brain. To clarify the impact that IF-treatment has upon aging, we used high throughput RNA-sequencing technology to examine the changing transcriptome in adult *Drosophila* tissues. Principle component analysis (PCA) and other analyses showed ~1200 age-related transcriptional differences in head and muscle tissues, with few genes having matching expression patterns. Pathway components showing age-dependent expression differences were involved with stress response, metabolic, neural and chromatin remodeling functions. Middle-aged tissues also showed a significant increase in transcriptional drift-variance (TD), which in the CNS included multiple proteolytic pathway components. Overall, IF-treatment had a demonstrably positive impact on aged transcriptomes, partly ameliorating both fold and variance changes. Consistent with these findings, aged IF-treated flies displayed more youthful metabolic, behavioral and basal proteolytic profiles that closely correlated with transcriptional alterations to key components. These results indicate that even modest dietary changes can have therapeutic consequences, slowing the progressive decline of multiple cellular systems, including proteostasis in the aging nervous system.

## 1. Introduction

Both human and model organism studies have shown even modest dietary modifications can have a significant positive influence on longevity and the health of aging individuals [1,2,3,4,5,6,7]. Existing research into the molecular mechanisms that underpin such improved health and longevity metrics indicates that the long-term function of multiple cellular systems is essential [3,5,8,9,10]. Indeed, numerous genetic studies have shown that a diverse range of regulatory, metabolic and clearance pathways are required for the healthy aging of the nervous system [8,11,12,13]. In addition, a wide range of environmental factors, especially modified diets, have consistently shown a positive influence on the age-related decline of select tissues as well as the overall functional health and performance of older individuals [2,10,14,15].

We have recently characterized a mild intermittent fasting (IF) protocol that promotes longevity and neural function of middle-aged adult *Drosophila* strains from divergent genetic backgrounds [1,16,17]. The beneficial effects of this fasting protocol included a delay in the progressive decline of locomotor behaviors and a reduction in neural aggregate levels [1,16,17]. This was associated with improvements in the acute and long-term functions of the autophagy pathway within middle-aged fly neurons [1,16,17]. Multiple studies have established that this pathway plays a central role in stress responses and in promoting healthy aging, partly through facilitating the clearance of protein aggregates and damaged cellular components [13,18,19]. At the mechanistic level, autophagy represents a downstream effector pathway, the activity of which is tightly regulated by multiple upstream factors that control and coordinate complex cellular responses [1,16,17,19,20]. The impact of IF-treatment on the aging CNS could reflect the alteration to multiple signaling and metabolic pathways that in turn influence pathway function, and as a result, the long-term maintenance of the aging nervous system [1,16,17,19,20].

Most organisms show well-defined aging phenotypes, which can reflect tissue-specific alterations to numerous molecular and metabolic pathways [15,21,22,23]. Taken together, these changes precede and eventually lead, to the functional decline (behavior, metabolism, stress responses), and the eventual death of an individual. Both genetic and drug-based studies using in vivo model systems have shown that the different genetic backgrounds and treatment regimens, which promote health and longevity, are often linked to basal changes to gene expression profiles [2,14,15,23,24]. Of note, a recent RNA-sequencing (RNA-seq) study that examined changes to the acult *C. elegans* transcriptome profiles, characterized a novel age-related phenomenon termed “transcriptional drift-variance” (TD), which reflects the progressive dysregulation of mRNA expression patterns [23]. In older worms, TD was significantly increased between replicate transcriptomes, which was partially suppressed in matching cohorts following exposure to the anti-aging compound, mianserin [23,25]. The analysis of transcriptome profiles from a range of mouse and human frontal cortex tissues samples also indicated that TD was an evolutionary conserved feature of aging [23]. A recent report examining the neural physiology of middle-age senescence-accelerated prone (SAMP8) mice also showed elevated TD levels in neural tissues. Following treatment with the neural protective compound, J147, TD levels were significantly reduced in mice and the average lifespan of adult *Drosophila* increased [26]. The implications from these studies are that environmental interventions that promote longevity, including IF-treatment, could facilitate more youthful mRNA expression patterns and variance profiles [1,2,5,26,27,28].

In this report, we examined the impact that aging and IF-treatment have upon the transcriptome profiles (non-fasted) in the adult *Drosophila* neural (head) and muscle (thorax) tissues [1,2,23,26]. Our analyses revealed that, by 4-week of age, both fly tissues showed significant basal changes to expression levels, with IF-treatment promoting more youthful global expression profiles. Both tissues exhibited the age-dependent phenomenon of transcriptional drift-variance, providing further evidence that the progressive disruption of transcriptional regulation also occurs in middle-aged flies [23,25,26]. Transcripts demonstrating fold changes and TD differences involved a wide array of genes representing a number of functional groups and pathways. In several cases, the age and IF-dependent changes were consistent with unique tissue-specific phenotypes and functional changes that included metabolic, behavioral and epigenetic systems [26]. Of particular interest were the dynamic variance changes, in neural tissues that occurred to multiple proteolytic pathway components, including members of the ubiquitin-proteasome system (UPS) and autophagy–lysosomal pathway. Globally these results indicate that even modest dietary manipulations could improve basal transcriptomic and phenotypic trends, which are themselves associated with partial suppression of several cellular processes linked to impaired cellular aging [23,26,29].

## 2. Results

### 2.1. Global Expression Differences Due to Age or IF-Treatment

To elaborate upon our original investigation into the impact of intermittent fasting (IF) on neuronal autophagy and aging, high throughput RNA-sequencing was used to examine global changes to the gene expression profiles in adult male *Drosophila* tissues. Examined were fly body segments that represented regions highly enriched with either neuronal (head) or skeletal muscle (thorax) tissues. Triplicate mRNA pools from adult heads were isolated from 1-week (1W), 4-week (4W) to 4-week IF-treated (4W-IF) male flies [1,20]. Matching thoracic mRNA pools (duplicates) were also isolated and used as reference non-neuronal control tissue set. Individual cDNA libraries were generated for each tissue and condition and then sequenced using Illumina HiSeq2000 technology [2,24,27,30]. Sequencing read mapping and alignments were done using the *Drosophila* reference genome (UCSC) and Genome Browser (genome.ucsc.edu, Santa Cruz, CA, USA), with sequence alignments and internal read mapping generated using TopHat and Bowtie (see Methods and Materials) [2,23,29]. Comparisons between annotated library sequences found that most reads (~80%) aligned with the reference transcriptome and represented ~94% exon-only sequences (Table S1).

**Figure 1:**
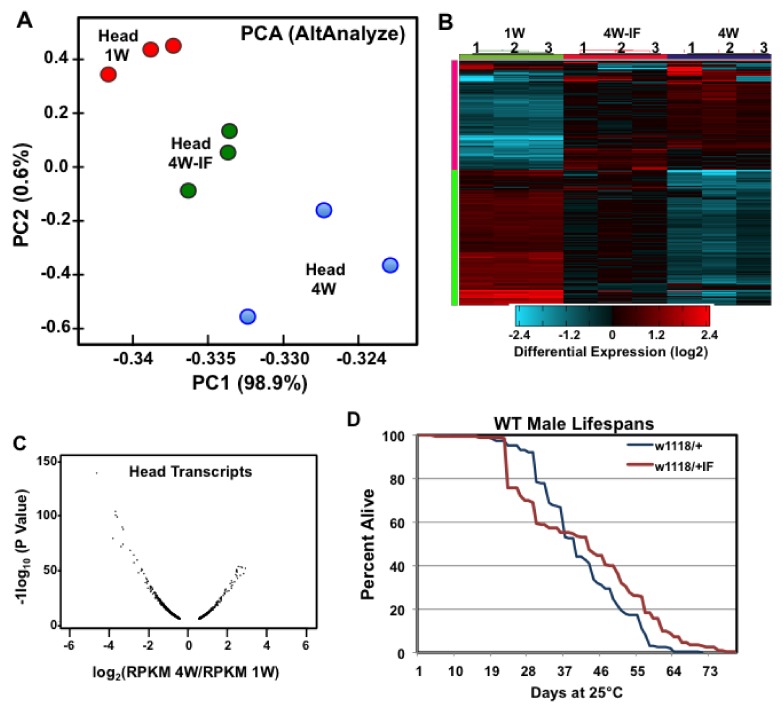
Principal component analysis (PCA) and expression clustering profiles of head transcriptomes. (**A**) The AltAnalyze software was used to compare PCA values for individual RNA-seq transcriptomes isolated from 1-week (1W), 4-week (4W) to IF-treated (4W-IF) male flies (*n* = 3). (**B**) AltAnalyzer was also used to establish the individual expression clustering profiles for head (*n* = 9) transcriptomes at different ages and treatment conditions. (**C**) Volcano plot showing significant message fold directional (±) changes in the CNS that occurs as a function of age (4W/1W, negative log10 of *p*-values as a function of log2). (**D**) Kaplan–Meier survival curves of male flies (*w^1118^*/+) maintained on *ad libitum* or IF conditions starting at 1-week of age (25 °C) [1].

As part of the predictive modeling of global transcriptome profiles, the high-dimensional variable analysis provided by the AltAnalyze analytical pipeline (Available online: https://www.altanalyze.org), was used to characterize the heterogeneity between individual RNA-seq datasets. This included performing principal component analyses (PCA) on total reads per kilobase of transcript, per million mapped reads (RPKM) values, which reduced the dimensionality of each expression dataset into two principal components (Figure 1) [23,29]. The PCA comparison of head transcriptomes demonstrated the close alignment between replicate samples as well as the expression differences that could largely be attributed to aging or IF-treatment (X axis, 98.9%, Figure 1A). PC analysis of head and thoracic datasets found distinct non-overlapping transcriptome profiles between the two tissues (PCA1 89.7%, Figure S1A), though both showed comparable shifts in global expression due to age or IF-treatment (PCA2 3.9%, Figure S1A). Interestingly, the head 4W-IF transcriptomes showed a significant tread toward more youthful patterns and had reduced TD variability when compared to matching non-fasted 4W controls (Figure 1A). Hierarchical clustering plots further highlighted the similarities between replicate samples as well as the impact that aging (4W) and IF-treatment (4W-IF) have upon global transcriptome trends (Figure 1B and Fgure S1B) [29,30]. The average RPKM values for transcripts typically expressed in muscle, neural or glial cell types were examined for both sets of tissues. In general, muscle specific genes were enriched in all thoracic samples (*n* = 6), while genes typically expressed in neural or glial cell types were preferentially detected across all RNA-seq samples from adult heads (*n* = 9, Table S2). This indicated that the transcriptome patterns from each body segment in large part reflected the expected tissue-specific expression profiles [2,29].

### 2.2. Age and IF-Dependent Changes in Transcriptome Profiles

The global assessment of neural profiles indicated that significant basal expression changes were occurring both as a consequence of aging and the IF-treatment regimen. Anticipating relatively modest changes to basal gene expression, the average fold change to each mRNA cohort was then examined, with significant cutoffs for aging set at >1.4 ± fold (4W/1W), and for IF-dependent changes, at >1.3 ± fold (4W/4W-IF) [2,29]. By 4-weeks, a similar number of genes from head (1197) and thoracic (1347) tissues showed substantial expression differences (Table 1). Age-related transcriptional changes in the fly head tended toward a broader deregulation of mRNA profiles (Figure 1C; Table 1), though the relative amplitude of such changes were more pronounced in 4W thoracic tissues (Figure S2A) [2,23]. In contrast, a larger number of neural transcripts showed a significant global response to IF-exposure when heads were compared to thoracic samples (294 versus 102 genes, Table 1). Further analysis of dynamic expression differences determined there was minimal gene overlap between fly head and thoracic tissues due to aging (Figure S2B; Table 1). Tissue-specific differences were also reflected in the IF-dependent responses, with neural tissues showing a significant shift toward more youthful expression patterns in 4W-IF cohorts, relative to those in the thorax (221 versus 58 genes, Table 1). The positive impact that IF-treatment has on progressive transcriptional changes in both tissues was consistent with the more general phenotype of enhanced longevity, which was demonstrated in our previous studies for male flies from several genotypes (Figure 1D) [1].

### 2.3. Gene Ontology (GO) or DAVID Analysis of Functional Pathway Changes

**Figure 2:**
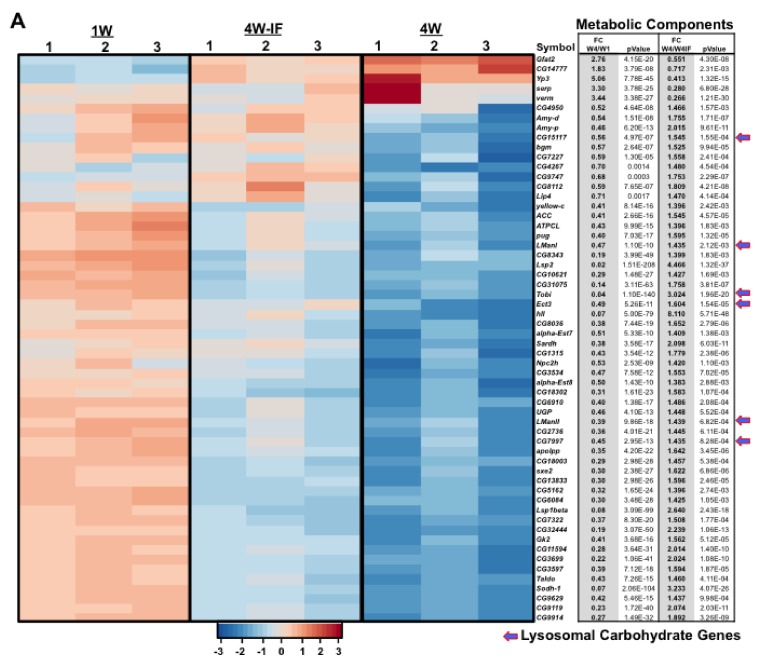

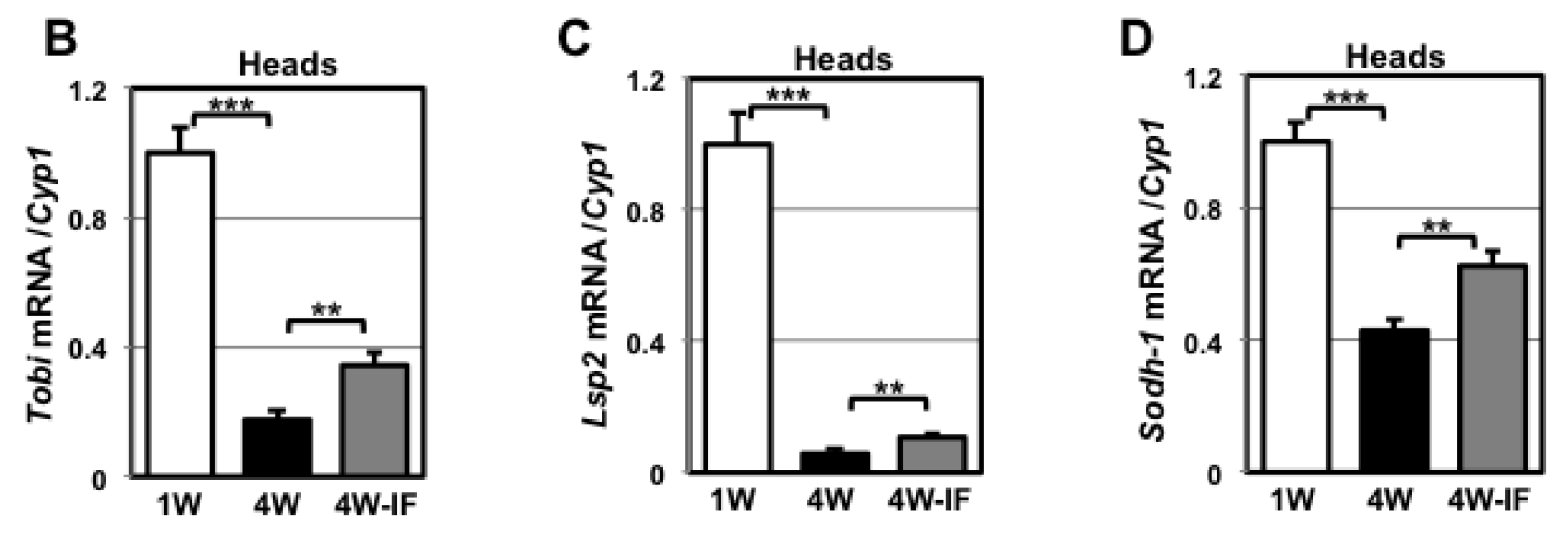
Age and IF-dependent fold changes (FC) to the expression profiles of metabolic pathway components in adult *Drosophila* heads. Quantitative RNA-sequencing and DAVID analysis identified metabolic genes that showed age and IF-dependent differences in expression levels. (**A**) Heatmap and corresponding table represents hierarchical clustering of scaled gene expression profiles and the respective fold change (FC) and *p* values between 4W/1W and 4W/4W-IF cohorts. Scaled expression values (*z*-score) were plotted in red–blue color scale with red indicating high expression and blue indicating low expression levels. Arrows highlight lysosomal proteins involved with carbohydrate metabolism. (**B**–**D**) qRT-PCR of expression changes to the *Tobi*, *Lsp2* and *Sodh-1* genes in neural tissues. ** *p* ≤ 0.01, *** *p* ≤ 0.001.

To assess the functional classification of genes or pathways influenced by age or IF-treatment, the DAVID bioinformatics program was used to identify functional clusters of genes with tissue-specific expression differences (Figure 2B; Table 1) [2,29]. For each condition, the transcriptome profiles from neural tissues showed a greater number of annotated functional groups with altered patterns of expression relative to thoracic tissues (Table S3). The largest functional gene clusters that showed dynamic age and youthful IF-dependent expression trends in the adult fly CNS were metabolic-related (Figure 2). Generally, these components are associated with longevity and lipid, carbohydrate (arrows) and mitochondrial related functions (Figure 2A). To confirm RNA-seq expression trends, quantitative RT-PCR (qRT-PCR) analysis of the *Tobi*, *Lps2* and *Sodh-1* genes was undertaken in neural tissues [31,32]. This indicated that the relative expression profiles of each gene matched the age and IF-dependent fluctuations detected by RNA-seq (Figure 2B–D). All three proteins have well-established metabolic functions and expression patterns that are influenced by aging, diet or alterations to the insulin-signaling pathway [31,32]. In thoracic tissues, the *Tobi* transcript also showed pronounced changes in expression due to aging and IF-treatment, albeit in the opposing directions (arrow, Figure S2C).

### 2.4. Age and IF-Dependent Neuronal and Behavioral Changes

**Figure 3:**
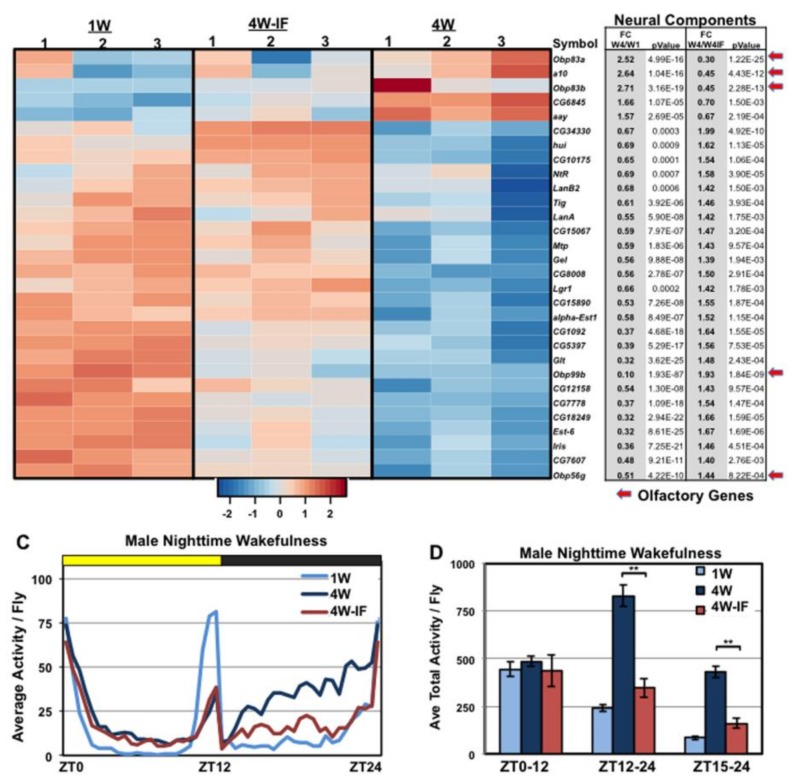
Age and IF-dependent expression changes to adult neuronal pathway components and adult olfactory-based behaviors. DAVID analysis identified genes with neuronal functions that demonstrated age and IF-dependent fluctuations in expression levels. (**A**) Heatmap and corresponding table represents hierarchical clustering of scaled gene expression profiles and fold change (FC) and *p* values between 4W/1W and 4W/4W-IF RNA-seq cohorts. Scaled expression values (Z-score) of RPKM expression levels were plotted in red (high) versus blue (low) color scale. Arrows indicate olfactory binding genes that are linked to behavioral defects. (**B**,**C**) Average 24 h activity profiles of grouped housed 1W, 4W and 4W IF male fly cohorts (*n* = 16 groups of 10 flies) and (**D**) Activity levels during light (ZT0 to 12) dark (ZT12 to 24) and mid-dark (ZT15 to 24) time periods. ** *p* ≤ 0.01.

Previously, we determined that IF-treated flies have lower insoluble protein aggregate profiles in neural tissues, which was linked to the preservation of adult climbing behaviors [1]. This was consistent with other modified diet studies that also detect a positive impact on the long-term function of neural tissues [1,33,34,35,36,37]. DAVID analysis revealed genes involved with a wide range of neural functions, including olfaction, had dynamic changes in expression patterns (Figure 3A). We had previously demonstrated that older male flies develop a marked increase in nighttime activity that was linked with the dysregulation of odorant binding protein (*Obp*) gene expression profiles and olfactory-based courtship behaviors [16]. In this study, elevated expression of *Atg8a* in neuronal tissues maintained both *Obp* expression patterns and suppressed the changes to courtship behaviors [16]. To determine whether IF-treatment could similarly alter nighttime activity profiles, 1W, 4W and 4W-IF treated male flies (*w^1118^*/+) were placed in group-housed conditions (10 flies/tube) and examined using 12-h light:dark (LD) cycling conditions for 48-h (LAM system). The 24-h activity profiles showed normal behavior patterns for young males (1W), including typical morning and evening peaks of activity, with extended mid-day and mid-dark rest periods (ZT15-21, Figure 3B,C) [16]. Middle-aged males (4W) demonstrated the normal increase in nighttime activity, which was largely suppressed in IF-treated cohorts (Figure 3B,C). Similar to transgenic enhancement of neuronal *Atg8a* levels, these results indicated that even modest dietary manipulations can protect the progressive dysregulation of this and other behaviors, further indicating the preservation of neural function in aged animals [1,16,17,19].

### 2.5. Altered Expression Patterns of Stress Response and Epigenetic Pathway Components

**Figure 4:**
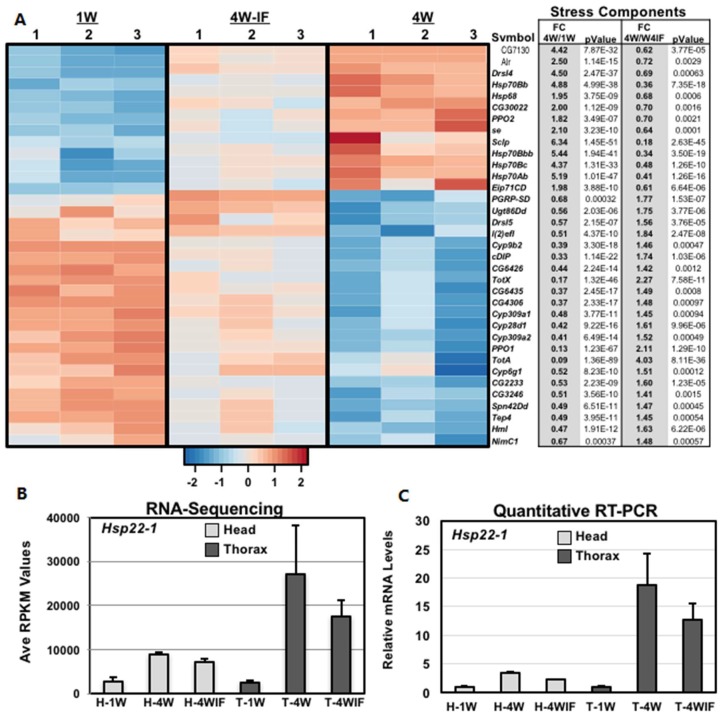
Age and IF associated changes to stress and inflammation pathway components in adult neural tissues. RNA-Seq and DAVID analysis of *Drosophila* head transcriptomes identified multiple genes in stress and inflammation related pathways that demonstrated both an age and IF-dependent difference in expression levels. (**A**) The heatmap and corresponding table represents the clustering of scaled gene expression profiles and includes the respective fold change in expression (FC, *p* values) that occur between 4W/1W and 4W/4W-IF tissue cohorts. Scaled expression values (*z*-scores) were plotted using a red–blue color scale represented dynamic differences expression differences to individual genes. Red indicates relative elevated expression and blue indicates relative reduced expression levels. The mRNA expression levels of *Hsp22-1* in head (**H**) and thorax (**T**) tissues measured in RPKM values by (**B**) RNA-seq or (**C**) qRT-PCR analyses. The relative mRNA expression of 1W flies (*w^1118^*/+) was set to 1.0 and subsequent RNA values were also normalized to the housekeeping *CXba* transcript.

**Figure 5:**
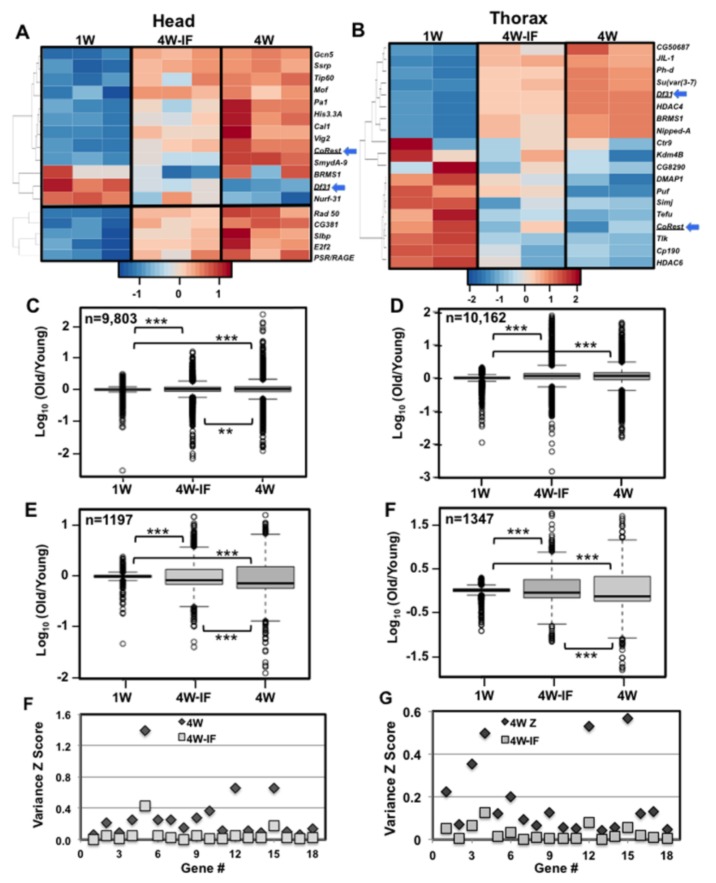
Age and IF-dependent changes to epigenetic pathway components and tissue specific transcriptional drift-variance profiles. Heatmaps of chromatin remodeling pathway components, showing dynamic FC to expression levels in aged (**A**) head or (**B**) thoracic tissue samples (n = number of genes). Arrows highlight the *Df31* and *CoRest* transcripts (blue arrows) that have significant but opposing tissue specific FC to expression profiles. Representative drift-plots showing the global variance changes or VC (~10,000 genes) to (**C**) head or (**D**) thorax tissue transcriptomes, isolated from 1-week 1W, 4W to 4W-IF adult cohorts. Drift plots representing (**E**) head and (**F**) thoracic transcripts (~1200 genes) that showed significant age-dependent FC to expression levels. *** *p* ≤ 0.001. See Table 2 for additional details. DAVID analyses were used to determine the functional pathways of head and thoracic genes that demonstrated elevated variance Z scores (4W/4W-IF VC > 3.75). (**G**) The scaled variance Z scores (SD/Ave) for individual transcripts involved with neuronal function were used to generate scatter plots of 4W (♦) and 4W-IF (■) head cohorts. (**H**) The scaled variance Z scores (SD/Ave) for lipid metabolic genes were plotted for 4W (♦) and 4W-IF (■) thoracic transcriptome samples.

DAVID analysis of thoracic samples identified significant fluctuations to the expression profiles of regulatory, proteolytic, vesicle transport and structural pathway components (Figure S3A–C). The fly CNS also demonstrated significant age and IF dependent fold changes to the expression levels of several stress response pathway components (Figure 4A). This included the *Drosophila Hsp22* transcript (mitochondrial heat shock protein), which in both head and thoracic tissues showed an age-dependent increase and IF-related decrease in expression levels (Figure 4B). Changes to *Hsp22* mRNA values were confirmed using quantitative (qRT-PCR) analyses (*n* = 16, Figure 4C), with expression patterns becoming highly variable in aged thorax samples (4W, Figure 4B,C). The age-related increase in expression variance for this gene suggested that transcription drift variance (TD) was occurring in older *Drosophila* tissues [23,26]. It also suggested that upstream transcription factors that regulated downstream expression and variance profiles could be influenced by protective dietary conditions [23,26]. Comparing the two tissues identified significant FC to the expression pattern of epigenetic pathway components, although there was minimal specific overlap between head and muscle samples (Figure 5A,B). For example the *CoRest* and *Df31* genes showed opposing response to aging IF exposure (blue arrows, Figure 5A,B). Taken together, our data suggests that both age and modified diets can produce unique tissue-specific changes to epigenetic pathway components in concert with the broader regulatory differences seen in the adult *Drosophila* transcriptome [38,39].

### 2.6. Age and IF-Dependent Changes to Transcriptional Drift

Neural PCA values (4-week, Figure 1A) and muscle *Hsp22* mRNA profiles (Figure 4B,C) suggested that middle-aged fly tissues showed an increased variability between matching replicate transcriptomes [23]. Therefore, the impact of aging and IF-treatment on transcriptional drift variance (TD) was assessed for both tissues. The global variance differences were examined for those genes with replicate RNA-seq values of >1.0 RPKM across all datasets for a particular tissues. The corrected log-fold change in expression variance was determined for each transcriptional cohort, using the 1W transcriptomes as ‘young’ reference values (log_10_[old/young reference_1W_]) [23,26,40]. Global TD profiles for head (9803) and thorax (10,163) mRNA cohorts are outlined in Table 2 and illustrated as drift-plots (Figure 5C–F, www.r-project.org) [23,26]. When compared to young flies (1W), total transcriptome profiles from aged cohorts (4W, 4W-IF) demonstrated a significant increase in expression variance or TD (Figure 5C,D) [23]. However, when the two aged profiles were compared, global TD levels were suppressed in 4W-IF neural samples (Figure 5C, Table S4). When a subset of genes that had age-dependent FCs in expression was examined, IF-treatment reduced the variance levels for both tissues (Figure 5E,F, Table S4). This analysis indicated that expression variance significantly increased in middle-aged *Drosophila,* while IF-treatment promoted more youthful TD or variance change (VC) profiles for both tissues, suggestive of alteration in global transcriptional regulation [23,26].

Further characterization of mRNA fold changes suggested that differences in expression patterns could be classified in terms of variability between replicate mRNA samples. Therefore, gene variance Z scores (**VZ**) from normalized replicate RNA-seq values were generated (VZ = SD/Ave RPKM). Comparisons were made between the different age (4W/1W) and treatment (4W/4W-IF) cohorts for both tissues, and individual genes with fold VCs were identified. Nearly 1300 neural and 1600 thoracic transcripts demonstrated a significant reduction in variance profiles following IF-exposure (4W/4W-IF VC > 3.75, Table 2). Often, gene specific FC to message levels did not coincide with VCs, which itself may reflect a heretofore-underappreciated feature of aging. DAVID analysis was performed on those transcripts in both tissues with high VC levels (4W), and that were suppressed in 4W-IF cohorts. In the aging CNS, genes with both FC and VC differences included odorant-binding and synaptic proteins and key components of the circadian pathway (*tim*, *per*) (Figure 5G, Table S5) [2,16]. Together with the age-dependent behavioral changes (Figure 3B,C), these finding suggest that examining both FC and VC differences could serve to clarify the genes and functional pathways that have malleable expression patterns that can be influenced by aging, diets or other modifying factors [23,26,29,41].

DAVID analysis was also performed on thoracic genes demonstrating age-related TD or expression variance patterns (4W/4W-IF). Several lipid pathway components showed substantial VCs that were significantly reduced following IF-exposure (Figure 5H), while the FC to expression levels remained largely unchanged (Table S6). As previously reported, basal and acute autophagy responses are influenced by age and IF-treatment, suggesting that whole animal fasting responses and rates of metabolic catabolism may also be similarly altered [1,2,32,42]. Therefore, the global starvation responses of 1W, 4W and 4W-IF treated flies were examined. Male flies were placed on fasting media (1% agar) and the number of dead flies counted every 8 h [43]. Middle-aged adults showed a heightened sensitivity to starvation that was partly suppressed in age matched IF-treated cohorts (Figure S4A,B). Since aged thoraces showed both substantial fold and variance changes to metabolic pathways, we examined the global catabolism rates of stored metabolites. Young (1W) and middle-aged (4W, 4W-IF) flies were fasted for 0 or 8 h, flash frozen and tissue homogenates used to determine whole body triglyceride (TG), glycogen and glucose levels (mg/mg protein, Figure S4C–H) [8,42]. Young flies had the highest basal stores and catabolism rates for all three metabolites (Figure S4C–H). Interestingly, older adults (4W) showed similar TG levels but lower consumption rates following and 8-h fast (14% decrease). In fasted 4W-IF cohorts, the TG catabolism profiles were more youthful (27.5% decrease, Figure S4C,D), while turnover of carbohydrate stores showed different profiles (Figure S4E–H) [42,44]. Together, this suggests that age and IF-dependent changes that are reflected as FC and VC expression differences in thoracic tissues have functional consequences in metabolic and survival responses in older individuals (Figure S4) [8,42].

### 2.7. Altered Proteolytic Pathways and Protein Aggregate Profiles in Aging Fly Tissues

**Figure 6:**
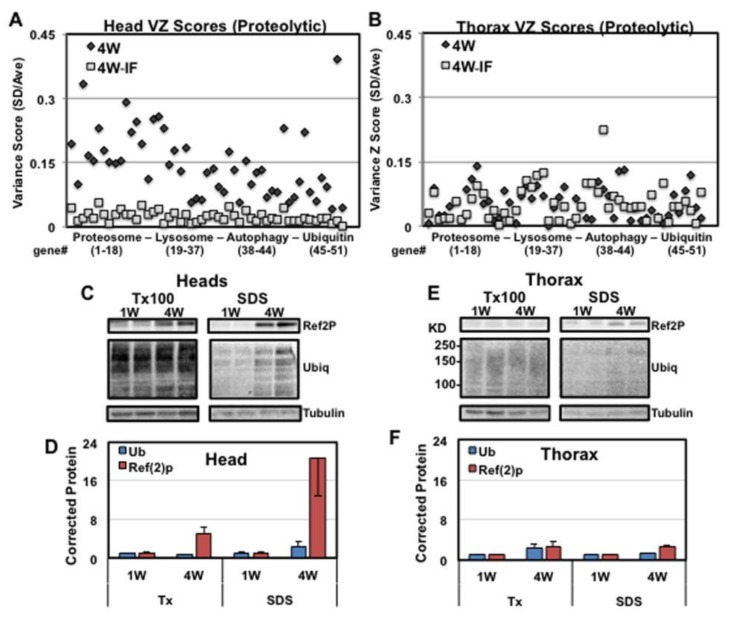
Age and IF-dependent tissue specific changes to proteolytic pathway variance and aggregate profiles. DAVID analysis was performed on neural transcripts that showed an age-related elevation and IF-reduction in TD variance scores (4W/4W-IF, >3.75). (**A**) Scatter plot of scaled variance Z scores (SD/Ave) of proteolytic pathway components from 4W (♦) to 4W-IF (■) head transcriptomes. (**B**) The scaled variance Z scores (SD/Ave) for the same cohort of genes from replicate 4W (♦) to 4W-IF (■) thoracic transcriptomes. See Tables S7 and S8 for individual gene information. Head and thoracic tissues from 1W to 4W flies underwent sequential detergent fractionation before Western analyses. Blots containing the Triton-X100 and SDS soluble protein fractions were probed with ant-UB, anti-Ref(2)P and anti-Tubulin antibodies and corrected protein levels quantified for both (**C**,**D**) head and (**E**,**F**) thoracic tissues. At 4 weeks of age, neural tissues demonstrated the buildup of protein aggregates, which is limited in age-matched thoracic samples.

Using a sequential detergent extraction and Western analysis, we have consistently shown that under normal culturing conditions that by middle-age, adult flies have a significant buildup of insoluble ubiquitinated protein (IUP) aggregates in neural tissues [1,16,17,19,45]. We have also demonstrated that the rate at which protein aggregates increase (IUP, Ref(2)P) closely correlates with the tissue specific decline in autophagic capacity, which can be manipulated by genetic, transgenic and dietary factors [1,16,17,19,45]. Close examinations of FCs found relatively few proteolytic pathway components with significant expression differences in either head or thoracic tissues (Figure S3B). In sharp contrast, DAVID analysis of neural genes with altered variance scores identified multiple elements in proteolytic pathways that became hyper-variable with age (4W), which were largely suppressed by IF-treatment (4W/4W-IF ratio > 3.5, Tables S7 and S8). Individually, this included proteasomal (18), lysosomal (21), autophagy (7) and ubiquitin (7) pathway components, which in neural samples had remained undetected due to minimal FCs to message levels (Figure 6A; Tables S7 and S8). Following IF-treatment (4W-IF) there was substantial suppression of variance profiles for the same set of genes (Figure 6A) [23,26,43,45,46]. In matching thoracic samples, these genes showed minimal FC or VC differences, indicating proteolytic pathway components were largely unaffected by aging or dietary conditions in muscle tissues (Figure 6B; Tables S7 and S8).

Given the marked tissue-specific differences in proteolytic expression patterns, the age-dependent buildup of aggregate profiles between head and thoracic tissue samples was directly compared. Sequential Triton-X100 (1%) and SDS (2%) detergent protein extracts were isolated from replicate 1W to 4W old fly heads and thoraxes and Westerns probed to establish protein aggregate profiles (Figure 6C–F). In neural tissues, Western blots of the SDS soluble protein fractions showed the normal age-dependent buildup of neural protein aggregates (4W) (Figure 6C,D). Age-matched thoracic samples showed minimal IUP and Ref(2)P level differences (Figure 6E,F). Together this was consistent with our studies showing the timeline and tissue specific development of protein aggregates in the aging fly CNS, which also coincides with altered transcriptional regulation for proteolytic pathway components [1]. These results also underscored the concept that therapeutic changes, including modified diets, can have a positive influence on a diverse array of tissue-specific cellular processes including proteolysis [1,23,26,29]. The transcriptional changes observed with the intermittent fasting based diet also appears to reflect the long-term positive consequences for aged individuals in terms of global and tissue-specific expression patterns and physiological responses [38,39].

## 3. Discussion

The progressive changes to multiple interconnected cellular systems has been mechanistically linked to the aging [1,8,16,39]. It has consequently been challenging to separate the primary cause(s) leading to the progressive deterioration of cells and tissues from the downstream or secondary collateral effects of aging [3,8,9]. Both genetic and environmental studies have established that multiple cellular pathways are involved with and can influence the normal aging process as well as long-term maintenance and function of the nervous system [1,12,16,25,34]. The implications from other longevity studies are that net positive changes due to diet likely involved subtle alterations to signaling, epigenetic, mitochondrial and proteolytic based pathways [2,3,25,28,31,47]. In this report, we have used RNA-seq analysis to continue our investigation into the dynamic expression and phenotypic changes that occur in adult *Drosophila* as a consequence of aging and exposure to intermittent fasting [1]. Previously, we have demonstrated that IF-treatment had a positive impact on middle-aged flies by slowing the progression of several global and neurodegenerative phenotypes [1]. This included longer average lifespans, the preservation of adult behaviors (geotaxis), lower neural aggregate profiles (IUP, Ref(2)P) and enhance basal and acute autophagic responses in the aging fly nervous system [1]. This implied that even modest dietary changes could have a significant impact on the regulation of cellular pathways and that are essential for the long-term functional maintenance of adult tissues.

For this study, the selection of mRNA samples (time, treatment, tissue) was based on well-defined age-dependent alterations to the behavioral and physiological profiles of wild type adult flies [1,17,45]. The over primary goal of these studies was to identify early changes to transcriptome profiles that could serve as the nexus to assess early changes to molecular mechanisms involved with progressive age-related defects, and to highlight cellular pathways that are responsive to environmental treatments [1,2,8]. Our initial comparisons of PCA and expression clustering profiles for individual RNA-sequenced transcriptomes confirmed the similarities between replicate samples, and highlighted the dynamic tissue-specific changes occurring to basal expression profiles as consequence of aging or IF-treatment (Figure 1 and Figures S1 and S2; Table 1 and Table 2) [29]. Further analysis confirmed that adult head and thoracic tissues have unique expression profiles that are selectively enriched for genes normally produced in neural, glial, or muscle cell types (Table S2). Global tissue-specific responses also included a relatively higher fold expression for thoracic transcripts, though neural tissues showed a larger number of genes responding to IF-treatment (4W/1W, Table 1). The relative number of genes that showed significant age-dependent FC and VC differences were similar between the two tissue types, although relatively few genes showed matching mRNA fluctuation patterns (Figure S2B).

IF-treatment may have a greater impact on the regulation of the adult head transcriptome profiles in part due to the level of tissue heterogeneity found within this *Drosophila* body segment. The majority of the adult thorax represents a relatively homogenous tissue, primarily comprised of flight and jump skeletal muscle cell type (see Table S2). Conversely, the adult fly head is comprised of diverse neural and glial cells, which have multiple functions and unique tissue specific transcriptional responses as a result of aging or IF exposure. The difference in the number of replicate transcriptome data sets used in our analysis of head (*n* = 3) and thorax (*n* = 2) tissues could have resulted in an under representation of expression differences between the two tissue types. However, the PCA and expression clustering analyses (Figure 1A,B and Figure S1) confirmed replicate sample similarities as well as the divergence expression trends that occurred as a result of age or dietary conditions for each tissue type. In addition, the relatively uniform number of genes showing age and IF-dependent changes between the two tissues (Table 1 and Table 2) suggested that analysis of duplicate RNA-seq data sets, while not optimal could be used to identify significant fold and variance changes to individual transcripts.

From these initial studies, non-directed DAVID analysis identified multiple functional groups and pathways that demonstrated unique tissue-specific FC in expression that could be attributed to both aging and nutritional conditions (Figure 2, Figure 3, Figure 4 and Figure 5 and Figures S2 and S3; Table 1 and Table S7) [23,29]. Each tissue showed unique age or IF-dependent changes to multiple pathway components, with only few genes demonstrating similar expression trends. One notable exception was the *Hsp22* gene, which in *Drosophila* serves as a chaperone protein involved with mitochondrial homeostasis, ROS detoxification and longevity-based functions [15,21]. Previously, fly tissues showed a basal age-dependent FC to *Hsp22* message and protein levels that closely correlates with enhanced longevity and stress resistance [15,21]. Consistent with these findings, RNA-seq and qRT-PCR analysis for both tissues showed an aged-dependent increase (4W) and IF-dependent reduction in the *Hsp22* message (Figure 4B,C). Together with elevated variance profiles in thoracic tissues (4W), this data suggests that IF-treatment reduces endogenous levels of cellular stress and may improve mitochondrial function in 4W-IF flies [15,21].

Recent studies examining the impact that aging and therapeutic compounds have on the regulation of adult *C. elegans* and murine transcriptome profiles, has highlighted the occurrence of transcriptional drift-variance in model organism [23,26]. This work has strengthened the concept that along with average FCs to mRNA levels, there exists an age-dependent increase in global expression drift-variance, likely reflecting the dysregulation of transcriptional stoichiometry [23,26]. Treatment with the drug mianserin (serotonergic pathway inhibitor) promoted longevity in adult *C. elegans* and resulted in a dose dependent reduction in TD and more youthful serotonergic pathway function [23,25]. Older mice exposed to the neural protective compound J147 also showed suppressed TD in neural tissues as well as the reduction in plasma metabolomics drift profiles [26]. Further, detecting TD variance in tissue transcriptome profiles from older human tissues suggests this is a conserved phenomenon, likely to involve progressive changes to epigenetic regulatory pathways [23,38,48,49]. In this study, we determined that tissue-specific changes to chromatin-remodeling pathway components (Figure 5A,B), closely correlated with the significant increase in TD profiles (4W) and the partial suppression of both following IF-treatment (Figure 5C–H). While both head and thoracic tissues showed a similar number of transcripts with an age-dependent increase in TD variance, the individual components were tissue-specific and largely did not overlap (Table 2). In addition, neural tissues appeared to have a greater response to IF-treatment and lower global expression variance profiles (4W-IF) than those found for matching thoracic transcriptome profiles (>3.75, Figure 5C–F; Table 2).

Since middle-aged *Drosophila* tissues showed unique VC transcriptional patterns DAVID analysis was used to characterized genes and functional pathways influenced by aging with IF-treatment and restored to more youthful pattern following IF exposure. In neural tissues genes showing lower VC have circadian (*tim*, *per*), olfactory (*Obp99a*), and neural (*neur, mnb, Fmr1, Sap47*) functions (Figure 5G; Table S5). The increase in TD profiles coincided with neuronal FC differences and the progressive decline and partial rescue of adult behaviors (Figure 3) [1,16]. For thoracic tissues, DAVID analyses revealed a very different set of genes showing dynamic FC and VC differences (Figure 5B,H and Figure 6B; Tables S6–S8). This included proteins involved with metabolism (Figures S2C and S4) as well as significant VCs to lipid homeostatic components (Figure 5H; Table S6) [32,42]. The functional potential impact of aging and IF-treatment on metabolic-based FC and VC in expression patterns closely correlated with improved starvation responses (Figure S4A,B) and more youthful catabolism rates for lipids (TG) in fasting 4W-IF flies (Figure S4C,D) [1,42]. Overall, the metabolic differences seen in IF-treated flies were consistent with previous dietary studies showing improved metabolic and physiological metrics in aged animals [2,32].

Along with age-related changes to key neuronal systems, the decline in CNS function is often associated with progressive proteostasis defects that include functional changes to the ubiquitin-proteasome and autophapy-lysosomal systems [1,16,17,25,34,50]. Indeed, the age-dependent formation of protein inclusions is often closely associated with the decline of motor behaviors, sensory perception (olfaction), sleep/circadian patterns and cognition [1,16,29,34]. Human studies and work using model systems have shown modified diets can slow the functional decline of the nervous system and may promote synaptic plasticity and neurogenesis in older adults [3,37,50]. In previous studies, we demonstrated that middle-aged flies exposed to IF-treatment demonstrated improved autophagy profiles including basal and acute fasting responses [1]. Consistent with this finding was the pronounced VC detected for multiple proteolytic components within the proteasome (*Rpn1,*), ubiquitin (*UBE4A*), lysosomal (*Cath-L*), and autophagy (*Atg16L2*, *PI3K59F*) pathways (Figure 6A; Tables S7 and S8). This appeared to be dynamic neural-specific VC, which was primarily not detected in matching thoracic samples (Figure 6B; Tables S7 and S8) [43,45,46,51]. Of particular note are key components involved with regulating proteostasis by the proteasome (subunits *Rpt*, *Rpn*) [46] and autophagy (*PI3K59F, Atg16L2*) [51], which showed significant VC and minimal FC in expression in contrasting transcriptome samples from aged fly cohorts (4W/4W-IF, Figure 6A; Tables S7 and S8). Taken together, the selective neuronal increase in proteolytic VC profiles was consistent with tissue-specific disparities observed in the timing and relative level of ubiquitinated protein aggregate accumulation (Figure 6C–F).

Overall this study indicates that conditions that alter in vivo aging phenotypes can be closely linked to expression difference to pathway components associated with individual functions. In addition, this analysis can also be used to identify upstream regulatory factors that coordinate the expression of key downstream functional components [39]. Therefore, observing the suppression of FC and TD profiles by select treatment conditions could not only highlight “sensitive” gene targets but also identify the cellular mechanisms that facilitate or promote their long-term regulation. This could include individual changes to signaling systems, transcription factors or epigenetic pathway components. In this study, IF-associated basal changes to expression patterns primarily highlighted differences to epigenetic components for both adult *Drosophila* tissues (Figure 5A,B). This implies that individual treatments or environmental conditions that alter aging phenotypes may be reflected as unique tissue-specific transcriptional “fingerprints” that are impacted by nucleosome and heterochromatin modifications [18,38,39,52]. In terms of healthy human aging, there is a growing appreciation that modest dietary interventions may be an effective method to preserve more youthful transcription patterns and facilitate the treatment of chronic progressive disorders [27,34,53,54]. The overarching goal for this type of treatment would be to maximize the benefit, while minimizing the side effects and compliance issues associated with harsher dietary regimens, including caloric restriction [34,54,55]. Given these attributes, applying targeted dietary modifications to aging population may have a lasting beneficial effect upon disorders linked to the functional decline of the nervous system [6,37,47,56,57,58].

## 4. Material and Methods

### 4.1. Drosophila Stocks, Culturing Conditions and Starvation Responses

Canton-S (CS) and *w^1118^* fly stocks (Bloomington *Drosophila* Stock Center, Bloomington, IN, USA), crosses and F1 offspring have been previously described and were originally obtained from the Bloomington Stock center (Bloomington, IN, USA) [1]. Flies used for each study represented F1 offspring generated from crosses between CS virgin females and *w^1118^* males (*w^1118^*/+) [16]. Adult male flies were collected within four hours of eclosion and maintained on standard fly media (25 per vial) and culturing conditions (25 °C, 65% humidity, 12-h light:dark, LD cycle) [1,16]. For IF-treatment studies, flies were maintained using standard conditions and media until 1-week of age, then were exposed to either IF or *ad libitum* culturing conditions [1]. IF-treated flies were turned onto fasting vials (1% agar) three times per week from 9:00 a.m. to 5:00 p.m. (8-h) [1]. For all studies, IF-treated flies were placed on *ad libitum* conditions for two full days prior to being used for behavioral and starvation studies or being flash frozen in liquid nitrogen for subsequent tissue isolation [1]. Flies were stored at −80 °C for before tissues were isolated and processed for mRNA, protein or metabolic analyses [1].

### 4.2. RNA Isolation, Library Construction, RNA-Sequencing and Bioinformatics Analyses

F1 fly cohorts (*w^1118^*/+) exposed to IF-treatment or *ad libitum* conditions for three weeks were collected and flash frozen at 1-week or at 4-week of age. *Drosophila* tissue processing, RNA isolation and cDNA library construction are outlined in Supplemental Information [16]. Libraries were sequenced using the Illumina HiSeq2000 technology (Illumina, Inc., San Diego, CA, USA) for single-end 100-bp format (7-base index). Briefly, staff at The Scripps Research Institute Sequencing Core Facility (La Jolla, CA, USA) processed sequencing data to generate FASTQ files demultiplexed based on index sequences [2,24]. Sequenced reads were mapped to a *Drosophila* reference genome (UCSC Genome and Browser (vmm9.gtf, UCSC, Santa Cruz, CA, USA) and alignments generated using TopHat (v2.0.9) and Bowtie 2 (v2.1.0, John Hopkins University, Baltimore, MD, USA) software., Cufflinks (http://cufflinks.cbcb.umd.edu/) was used to assemble sequence reads for individual transcripts [2,24,29]. For each sequenced library, the number of reads per gene was normalized and reported as RPKM values as an estimation of expression levels. To determine the fold changes, both the average RPKM and log_2_ RPKM scaled values for each gene was determined from replicate reads (FC = Ave log2 RPKM 4W/Ave log2 RPKM 1W). Significant expression differences due to aging were set at >1.4 (4W/1W) and IF-treatment at >1.3 (4W/4W-IF). In addition, normalized RPKM values used to generate heat maps (fold), variance fold changes and drift-variance plots (box plots) from different transcript cohorts and are further detailed in Supplemental Information. The AltAnalyse software (v2.1.0, Cincinnati Children’s Hospital, Cincinnati, OH, USA) was used for PCA analysis and to generate expression-clustering profiles for each RNA-seq data set. The DAVID bioinformatics program (https://david.ndifcrf.gov) was used to identify functional gene clusters and associations for transcripts showing fold or variance differences due to age or IF. A custom designed Python program (Python 3.6.4, Copyright© 2001–2018 Python Software Foundation, https://www.python.org/psf/) was used to refine functional pathway gene lists that were used to generate heatmaps, expression tables and variance scatter plots. Methods used to normalize values for heatmaps, transcriptional drift and drift-variance profiles and normalized drift-variance values are further detailed in Supplemental Information.

### 4.3. 24-h Activity Profiles of Aged Group-Housed Male Flies

Outcrossed male flies were collected within 4 h of eclosion and aged in cohorts of 25 using standard husbandry and IF conditions. Flies were entrained using 12-h light and 12-h dark conditions (LD), with lights-on starting at 8:00 a.m. and lights-off at 8:00 p.m. [16,20]. The activity profiles of group-housed male flies were examined using the LAM25 systems (Trikinetics Inc., Waltham, MA, USA) and established protocols [16]. The LAM25 system detects fly movement (infrared beam breaks) and activity events were detected using the DAM System3 program (Trikinetics Inc., Waltham, MA, USA) [16]. Following an overnight recovery, the activity profiles of 1W, 4W and 4W-IF male fly groups (10 per vial) were monitored for 48 consecutive hours, using 12-h LD conditions [16]. Collected data sets were further analyzed using a custom-designed Python program (https://www.python.org/psf/, Python v3.6.4, Copyright © 2001-2018 Python Software Foundation) and Microsoft Excel software (Microsoft Office^®^, Mac 2011 v14.7.1) to generate activity graphs during the 12-light (ZT0–12), 12-dark (ZT12–24) and 6-h mid-dark (ZT15–21) time periods [16].

### 4.4. Quantitative RT-PCR

Flies from different ages and treatment conditions were collected, flash frozen and stored at −80 °C. Head and thoracic tissues were isolated, cDNA libraries generated quantitative Real-Time PCR studies performed using established technique [16,20]. The relative mRNA expression of 1W WT flies (*w^1118^*/+) was set to 1.0 and subsequent RNA values were also normalized to the housekeeping *CXba* transcript [1,16,19]. Primer sequences will be provided upon request and additional methods details are included in Supplemental Information.

### 4.5. Metabolite Profiles

Adult male flies from 1W, 4W, or 4W-IF conditions were fasted for 0-h, 4-h or 8-h on 1% agar prior to final collection, flash freezing and storage at −80 °C. See Supplemental Information for additional detailed methods used to determine whole body triglyceride, glycogen and glucose levels and Figure S4 for results [1,42,43].

### 4.6. Western Blot Analysis

Outcrossed male flies were aged, collected and flash frozen at 1 and 3-week of age. Adult heads and thoraces were collected and individual tissues homogenized using standardized reagents and techniques. [1,16,20]. See Supplemental Information for detailed methods.

### 4.7. Statistical Analysis

Quantified Western blot, activity profiles, and metabolic graphs were generated using Microsoft Excel and figures assembled in PowerPoint (Microsoft Office^®^, Mac 2011 v14.7.1) [16]. Unless otherwise stated statistical analyses between groups were performed using the GraphPad software (https://www.graphpad.com/) and Student’s *t*-test (two-tailed, unpaired). All values are reported as mean values ± SEM [16,20].

## 5. Conclusions

This and previous studies have demonstrated that even modest dietary modifications can have a significant beneficial impact on the cellular processes and physiological responses of aging worms, flies, mice and humans [1,2,3,5,47]. Along with promoting longevity and healthy neural aging, we have shown that IF-treatment can have a net positive impact on the progressive decline of several additional phenotypes that are reflected by the tissue-specific fluctuations to adult *Drosophila* transcriptome profiles [1,2]. This includes more youthful metabolic responses, the maintenance of olfactory-based courtship behavior, and the neural specific buildup of proteins aggregates [1,3,4,7,16,34]. The work detailed in this study indicates that even modest dietary changes can have a significant impact on progression of aging phenotypes, which in turn is reflected by significant differences in basal expression patterns. Our results indicate that protein homeostasis and clearance pathways in neural tissues are influenced and that changes to upstream epigenetic factors may be mediating these responses [59,60].

## Figures and Tables

**Table 1:** Genes with Fold Changes due to Aging and Intermittent Fasting.

Tissue	Change with Age	Age Down	Age Up	Change with IF	Age Down IF Up	Age Up IF Down	IF More Youthful
Thorax	1347	790	557	102	22	36	58
Head	1197	806	391	294	178	43	221

**Table 2:** Variance Changes * with Age and IF.

Variance > 3.75	4W/1W	4W/4W-IF	1W/4W-IF
Thorax genes	1457	1592	1441
Head genes	1156	1322	520

* Based on replicate RPKM values and corrected Z scores (SD/Ave).

## Supplementary Materials

The following are available online at http://www.mdpi.com/1422-0067/19/4/1140/s1.
