# Supplementary material for: Aging and Intermittent Fasting Impact on Transcriptional Regulation and Physiological Responses of Adult Drosophila Neuronal and Muscle Tissues
Source: Int J Mol Sci. 2018 Apr 10;19(4):1140. doi: 10.3390/ijms19041140 (PMC5979431; doi:10.3390/ijms19041140)
Supplement: Supplementary file 1 [file ijms-19-01140-s001.pdf]

International Journal of
Molecular Sciences

MDPI

Supplemental Information

Experimental Procedures

Tissue isolation, RNA library construction and RNA sequencing: Fly cohorts at 1-week and 4-weeks of age, exposed to IF treatment or *ad libitum* conditions were collected, flash frozen and stored at -80°C [1, 2]. Heads were collected by vortexing frozen flies, quickly passing severed heads through a tea sieve and collecting individual cohorts (~100 heads per replicate, age and treatment condition) [3, 4]. While on dry ice, individual thoraxes were dissected away from other tissues and stored at -80°C for subsequent RNA isolation (~50 per replicate). Tissues were homogenized using Omni Bead Rupter-24 system (Omni International, Inc., Kennesaw, CA, USA) and total RNA extracted using TRIzol™ LS reagents and protocols (ThermoFisher Scientific, Inc. Carlsbad, CA, USA) [3-5]. RNA quality was assessed using Nanodrop Spectrophotometer 2000 System (ThermoFisher Scientific, Inc. Carlsbad, CA, USA) and Agilent Bioanalyzer (Agilent, Santa Clara, CA, USA) [6, 7]. For each sample approximately 500 ng of total RNA was depleted of ribosomal RNA using RiboMinus™ reagents and techniques (ThermoFisher Scientific, Inc. Carlsbad, CA, USA) [8]. 100 ng of depleted RNA was used with the TruSeq RNA Sample Prep Kit to construct paired-end sequencing libraries following the instructions in the TruSeq RNA Sample Preparation V2 Guide (Illumina Inc., San Diego, CA, USA), following manufacturer's recommend protocols and 15 PCR cycles [8-11]. Final libraries were size selected on 2% agarose gels to obtain product lengths between 280-380 bases. Each library was loaded onto an Illumina HiSeq2000 for single-end 100-bp reads with 7 bases of the index read.

Determination of RPKM estimation: Data was processed to generate FASTQ files using CASAVA 1.8 and demultiplexed based on index sequences [8-11]. After read mapping, transcripts were assembled using Cufflinks software (version 1.3.0). To facilitate downstream analyses (such as gene fold change analysis, etc.) and to assess the accuracy and reliability of each RNA-Sequencing reads, a threshold for reliable RPKM estimation was employed based on optimizing the intersection of false-positive and false-negative rates [12-14]. The analysis was based on the 95% confidence intervals of RPKM values calculated by Cufflinks [13, 15]. Genes with RPKM values with lower confidence bounds of 0 were determined as "unreliable" and for most analyses RPKM values of 1.0 or greater were set as the threshold minimum for each expressed gene for subsequent analyses. Sequencing data sets were processed to generate FASTQ files using Illumina CASAVA-1.8 filter and genome alignments preformed using TopHat 2 (v2.0.9) and the *Drosophila melanogaster* reference genome (UCSC, Santa Cruz, CA, USA) [10, 11, 16].

EdgeR was used to determine the changes (**FC**), Magnitude Fold Changes (**Magn FC**), logCPM, P-values and false d number of Differentially Expressed transcripts (**DE**) from the average RPKM values of transcriptomic data sets for each cohort. Subsequent filtering and reporting of data sets were done using Microsoft Excel [10, 11, 16]. Sequencing data was processed using AltAnalyse software (v2.1.0, Cincinnati Childern's Hospital, Dr. Nathan Salomonis, Cincinnati, OH, USA) to generate Principal Component Analyses (PCA) and expression clustering profiles of RNA-seq data sets. Volcano plots were used to illustrate tissue-dependent Fold changes and were calculated based on mean RPKM values from replicate 1W, 4W and 4W-IF thorax and head samples [7, 17, 18]. Each gene was represented by individual dots with significant directional fold changes in (≥1.4+/-) in expression levels. The $\log_2(RPKM_{W4}/RPKM_{W1})$ values were plotted against the $-\log_{10}$(P-values) for each gene and tissue type. The Venn diagram of overlapping gene expression profiles was generated using Microsoft PowerPoint [17, 19]. The online Database for Annotation, Visualization and Integrated Discovery (***DAVID*** 6.8; https://david.ncifcrf.gov) bioinformatics resource was used to identify and annotate functional gene groups that showed significant expression or variance changes as a function of age or IF-treatment [20-22].

Normalized expression values for heat maps: To illustrate changes in expression profiles, individual RPKM values were transformed to $\log_{10}$ scale values. Averaged $\log_{10}$ RPKM is calculated for each gene [Ave=average ($\log_{10}$RPKM 1W, 4W, 4WIF)], and this served as mid-point or "0" value.

Z-score measuring how far expression level is from the mid-point were generated for each replicate and each biological condition [Z = (log_{10}RPKM 1W, 4W, 4WIF) - Ave] [7, 17, 18]. A positive Z-score means a given expression is above the mid-point (red), and a negative Z-score means a given expression is below the mid-point (blue) [18]. Heat maps were made using color theme "RdBu" from the R ColorBrewer and gplot packages via the heatmap.2 function [7, 18].

Establishing transcription drift-variance profiles: The *Drosophila* RNA-sequencing data was analyzed for age-dependent changes to variance profiles that represented changes to 'transcriptional drift' (**TD**) with respect to variance profiles [17, 18, 23]. TD was defined as the change in gene transcript RPKM values between young flies (1W, young reference) and aged flies represented as age (t) (4W or old fly cohorts).

Equation 1 normalizes the transcription levels for all genes (RPKM) to 0 against young 1W fly values.

Equation 1 $td_{gene\ X} = \frac{\log(\text{No. of transcripts}_{age(t)})}{\log(\text{No. of transcripts}_{young\ reference})}$ or $td\ gene\ X = \left(\frac{\text{RPKM}_{age\ (t)}}{\text{RPKM}_{young\ ref}}\right)$.

In case multiple replicate data-sets of the "young reference" were available, we incorporated multiple "young reference" data-sets by calculating a mean gene expression level to generate the "young reference' values for each gene, gene (x).

Equation 2 Log (No. of $\text{transcripts}_{young\ reference}$) = Ave [log (No. of $\text{transcripts}_{young1,\ young2,\ young3}$)] values. This method was used to calculate the average "young reference" fly cohorts at 1W of age. The advantage of this method is that the results for all subsequent ages is more robust as several "young reference" samples are included and thereby reducing the overall noise. Each replicate of the tissue-specific "young" cohorts were compared with averaged "young reference" [17, 18, 23].

Equation 3 was used to calculate the *drift variance* = $\frac{1}{n-1}\sum_{i=1}^{n}(\text{td}\ i - \text{td})$ for different RNA-seq sample cohorts. To evaluate changes in co-expression, we calculated the drift-variance (dv) over a group of n genes with transcription drift ranging from $TD_{i=1}$ to $TD_{i=n}$, where **n** is number of genes [7, 17, 18]. Genes that maintain a close co-expression patterns between replicate samples show drift-variances that are relatively small. Depending on whether a large fraction of genes within a transcriptome have change opposing directional changes to expression patterns, the increase in drift-variance suggests a loss of youthful co-expression patterns. To establish statistically different drift variances, the Wilcoxon Rank Sum test used R software [7, 17, 18].

Global Drift-variance profiles and individual gene variance values: The global and subdivision of transcriptional drift variance is illustrated as boxplots. For all age and treatment cohorts, each gene data point was converted into a Z-score ($\text{Z-score}_{gene\ X} = TD_{gene\ X}$) [7, 17]. The boxplots for each tissue, age or treatment RNA-sequencing cohort represented the minimum, first quartile, median, third quartile, and maximum TD distributions and were generated using R software [7, 17]. The individual gene variance value was used to identify hyper-variable expression patterns, the gene specific TD Z-scores (RPKM STDEV/Ave) were generated for all replicate RNA-seq cohorts representing individual fly tissues, ages or treatment conditions. Variance changes between different ages and treatment conditions (*i.e.* VC = Z-4W/Z-4W-IF) were also generated for each gene. Genes showing VC scores of >3.75 between 4W and 4W-IF RNA-seq cohorts were selected for DAVID analysis to identify genes and pathways that had variance differences influenced by age or IF-treatment (enriched annotation cluster scores ≥1.3) [20-22]. Gene specific drift-variance of 4W and 4W-IF tissue specific cohorts and expression fold changes (**FC**) were graphed using Excel [7, 17]. The actual Z-scores (RPKM STDEV/Ave) of genes from functional groupings showing a significant age and IF dependent changes to expression variance profiles were illustrated as scatter plots using Excel software.

Quantitative RT-PCR: Flies from different ages and treatment conditions were collected, flash frozen and stored at -80°C. Triplicate mRNA extractions (Trizol) and cDNA libraries (25 heads) were prepared for each fly genotype and age [2-4]. The RevertAid First Strand cDNA Synthesis kit and a mixture of random hexamer and oligo-dT primers were used to generate cDNA libraries (Thermo Scientific, Pittsburg, PA, USA) [3]. A CFX Connect Real-Time PCR Detection System (Bio-Rad, Hercules, CA, USA), transcript specific primers and SensiMix SYBR kit reagents (Bioline USA Inc., Taunton, MA, USA) were used to obtain qRT-PCR data from replicate cDNA libraries assayed in

triplicate using the Pfaffl method [2-4]. Relative 1W expression levels served as a reference value for each message (1.0) and subsequent values were normalized against *EXba* transcript [2, 3]. Primer sequences for individual genes are available upon request.

Starvation and Fasting Responses: Flies were flash-frozen and stored at -80°C and used to isolate tissue-specific mRNA pools or for protein and metabolic analyses. To assess starvation responses, male flies at 1W, 3W or 3W-IF were placed in vials containing 1% agar (wet starvation) and the number of dead flies counted every 8-hrs and used to establish average lifespans (hours) [1, 24].

Metabolite Levels: Analysis Adult heads were removed to prevent interference of the eye pigment for colormetric assays. For each condition, replicate samples (10 bodies) were homogenized in 30 µl PBS, vortexed and centrifuged for 10 minutes at 4°C (14,000 rpm). For triglyceride analysis, 5 µl of fly homogenate was combined with 200 µl of Infinity Triglyceride Reagent (Sigma-Aldrich, St. Louis, MO, USD) in triplicate, along with triglyceride standards (StanBio, Boerne, TX, USA) [25, 26]. This assay detects all the forms of glycerides (tri-, di-, and mono-) that represent 90% of insect lipids [25, 26]. Glycogen and glucose levels were also determined from adult tissue homogenates (10 bodies in 60 µl buffer, 10 mM KH_2PO_4, 1 mM EDTA, pH 7.4). Glycogen was first reduced to glucose using a solution containing amyloglucosidase (Sigma-Aldrich) and then glucose levels were measured using a method used to determine blood glucose [27, 28].

Briefly, glucose oxidase/peroxidase (PGO, Sigma-Aldrich) and o-Dianisidine solution (Sigma-Aldrich) was made in 20 ml of water as previously described [27, 28]. Replicate tissue samples and glycogen standards (Sigma-Aldrich) were added to each well together with 220 µl of glycogen assay solution and incubated for 30 min at 37°C and read at 450 nm. To determine changing glucose levels, 3.0 µl of each homogenate was combined with 200 µl of Infinity Glucose Reagent (Sigma-Aldrich) in a 96-well plate format [27, 28]. Glucose standards (StanBio) were included on each plate, incubated at 37°C for 10 minutes and read at 500 nm using a Molecular Devices SpectraMax spectrophotometer and linear regression used to determine the standard curve. Each experiment was repeated at least three times. The decline in TG, glycogen and glucose levels and the percent remaining were determined from 0 and 8-hr fasting condition values. Protein levels were determined for each sample using 2.0 µl of fly extract, 4.0 µl of PBS and DC Protein Assay reagents and bovine serum albumin standards, following manufacturer's instructions (BioRad) and measured at 750 nm [1, 27, 28]. The protein concentration for each sample was used to normalize whole body triglyceride, glycogen and glucose values.

Western Blot Analysis: For sequential detergent extraction, adult fly tissues were initially homogenized in a Triton X-100 based buffer (1.0%) followed by extraction in a SDS (2.0%) buffer as described previously [1, 4, 5]. Protein concentrations for each sample were determined using the DC Protein assay (Bio-Rad). Separated tissues were extracted with lysis buffer (2% SDS, 150 mM NaCl, 50 mM Tris, pH 7.5) containing protease inhibitors (Thermo Scientific and Pierce, Rockford, IL, USA) using the Bead Ruptor-24 System (Omni International). Cellular debris was removed (10,000x*g* for 10 min) and the protein concentrations of supernatants were determined using the DC Protein assay (Bio-Rad). Protein samples (20 µg) were resolved on a 12% Bis-Tris gel (Bio-Rad) and transferred onto PVDF Immobilon-P membranes (Millipore Corp., Billerica, MA, USA) using the Trans-Blot Turbo system (Bio-Rad). Blots were sequentially probed using anti-Ref(2)P (Ratliff et al., 2015), anti-Ubiquitin (P4D1, Cell Signaling Technologies, Danvers, MA, USA) and anti-Actin (JLA20, Developmental Studies Hybridoma Bank, Iowa City, Iowa, USA) antibodies at various dilutions overnight at 4°C [1, 4, 5]. Blots were developed using Thermo Scientific West Dura Substrate (Thermo Scientific/Pierce) and the ChemiDoc digital Imaging System and Quantity One software (Bio-Rad). Protein band intensities were quantified using ImageJ software (https://imagej.nih.gov/ij/) [1, 4, 5].

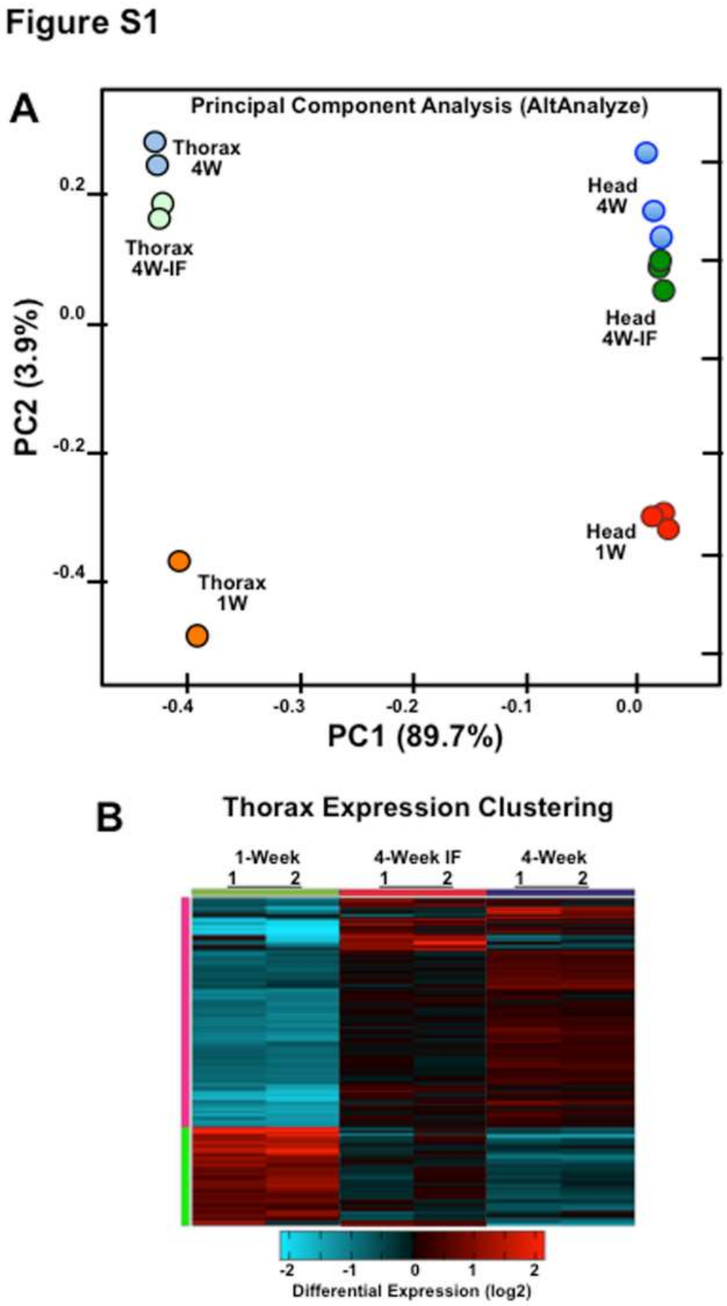

Figure S1. Principle component analysis and expression clustering profiles of adult *Drosophila* thoracic transcriptomes. A) AltAnalyze was used to compare individual PCA values for RNA-seq transcriptomes of thoracic tissues isolated from 1-week (1W), 4-week (4W) and IF-treated (4W-IF) male flies (n=2). **B**) AltAnalyzer was used to establish the individual expression clustering profiles for individual thoracic (n=6) transcriptomes at different ages and following different treatment conditions.

Figure S2

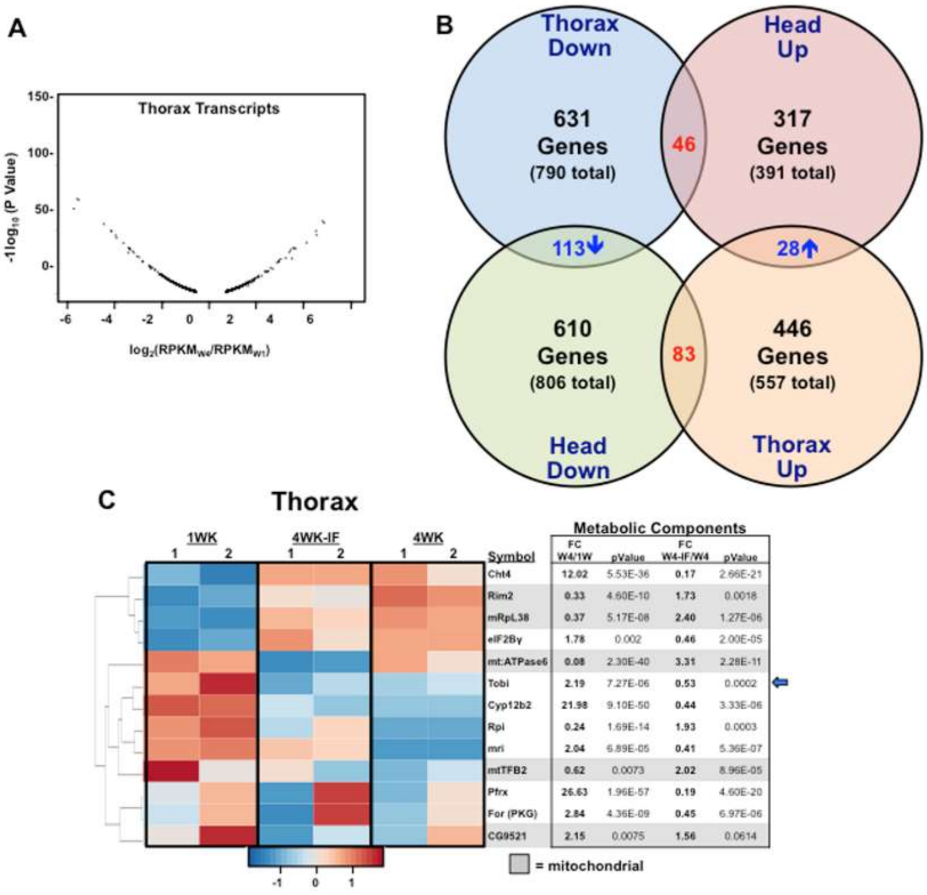

Figure S2. A) Volcano plots show the significant directional (+/-) changes to fly transcripts (negative $\log^{10}$ of P-values as a function of $\log^{2}$) that occur as a function of age (4W/1W). **B**) Venn diagram of genes showing significant reductions (down) or increases (up) in expression profile in adult tissues (4W/1W). Also noted are the numbers of genes with similar (blue) or opposing (red) tissue-specific expression trends. **C)** Heatmap and table showing age and IF-dependent changes to metabolic pathway components. Arrow indicates the Tobi gene expression profiles and gray highlights changes to mitochondrial genes.

Figure S3.

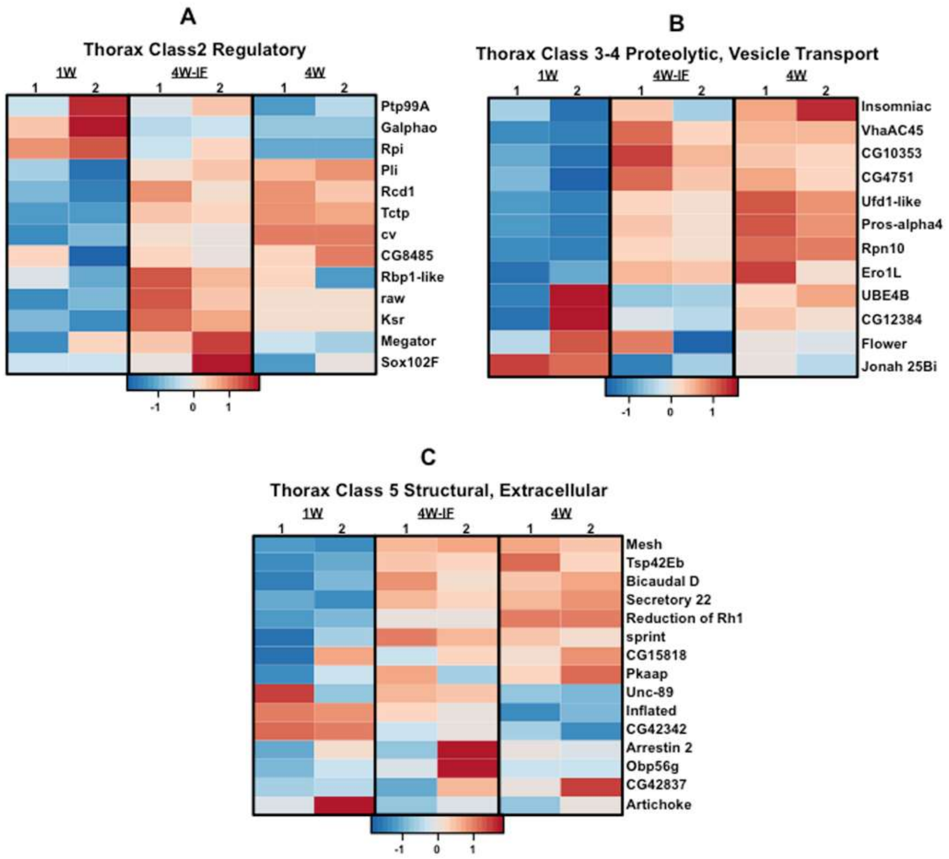

Figure S3. Age and IF-dependent expression changes occurring in adult thoracic tissues. Quantitative RNA-Seq and DAVID analysis identified subsets of genes that have altered expression profiles in thoracic tissues. Heatmaps represent scaled expression values (Z-score) for individual 1W, 4W-IF and 4W sequencing reads, plotted as red–blue color scale with red indicating elevated and blue indicating reduced expression levels. Heatmaps primarily represent functional gene groupings that are associated with **A**) regulatory, **B**) proteolytic, vesicle transport, or **C**) structural and extracellular matrix pathway functions.

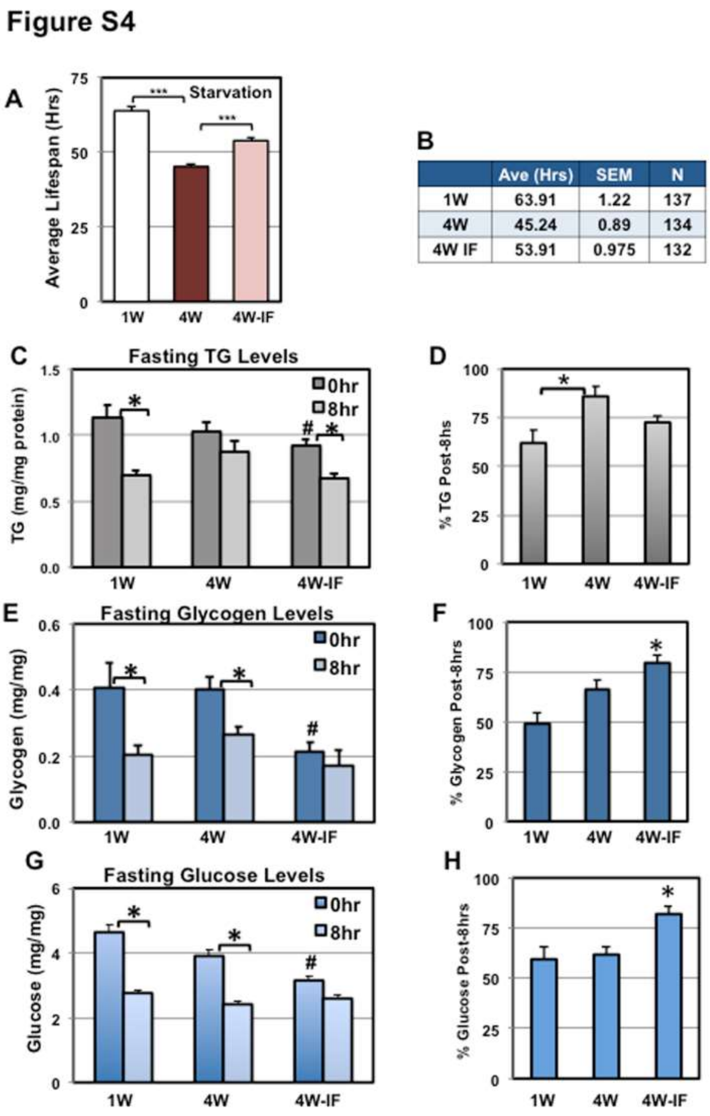

Figure S4. Changes to the starvation responses, basal triglyceride and carbohydrate profiles and catalytic rates of adult *Drosophila*. A-B) The lifespan average profiles of 1W, 4W and 4W-IF treated adult flies exposed to starvation conditions or a hard fast. The number of dead flies for each cohort were recorded every 8-hrs (hours). C-D) Whole body levels (without heads) of triglycerides (mg/mg protein) from control 1W, 4W and 4W-IF adult male flies and following an 8hr fast. D) The percentage of triglycerides remaining following an 8hr fast (8hr/0hr values) in different fly cohorts. E-F) The glycogen and G-H) glucose (mg/mg protein) profiles were also determined following a 0hr and 8hr fast, for adult male flies at 1W, 4W and 4W-IF of aging. The percentage of remaining D) glycogen and F) glucose remaining following an 8hr fast (8hr/0hr) was determined for 1W, 4W and 4W-IF adult non-neuronal tissues #,*P≤0.05, ***P≤0.001.

Table S1. Summary of RNA Sequencing Reads

Samples	Total Number of Reads	% Exon Only Reads	% Partial Exon Reads	% Intron Only Reads	% Non-Gene Reads
1WK Head 1	18,547,546	94.2	1.17	1.73	2.9
1WK Head 2	16,656,520	94.6	1.12	1.73	2.6
1WK Head 3	17,087,497	94.5	1.13	1.78	2.5
4WK Head 1	14,276,548	94.2	1.25	1.73	2.8
4WK Head 2	15,091,766	94.5	1.21	1.78	2.5
4WK Head 3	13,933,185	94.7	1.17	1.76	2.4
4WK Head IF 1	14,367,159	94.9	1.16	1.73	2.2
4WK Head IF 2	13,958,530	95	1.12	1.66	2.3
4WK Head IF 3	17,345,308	95	1.15	1.56	2.2
1WK Thorax 1	24,258,130	94.7	1.23	1.39	2.7
1WK Thorax 2	18,920,727	94.6	1.18	1.43	2.8
4WK Thorax 1	21,238,932	95.5	1.17	1.28	2.1
4WK Thorax 2	16,937,732	95.9	1.09	1.17	1.8
4WK Thorax IF 1	19,807,804	95.3	1.29	1.43	2
4WK Thorax IF 2	21,203,458	95.7	1.17	1.33	1.8

Table S2. Average Tissue-Specific RPKM Values

Gene (ID)	Tissue	Average	SEM
ELAV	Head	24.04	0.88
FBgn0260400	Thorax	9.33	0.22
Repo	Head	26.47	0.73
FBgn0011701	Thorax	12.24	5
Synapsin	Head	77.98	1.89
FBgn0004575	Thorax	26.01	10.62
Oct-TyrR	Head	5.45	0.17
FBgn0004514	Thorax	1.98	0.81
Neurexin 1	Head	64.43	2.54
FBgn0038975	Thorax	15.62	0.69
Neuroligin 2	Head	21.27	0.42
FBgn0031866	Thorax	3.44	0.25
Neuroligin 4	Head	8.95	0.31
FBgn0083975	Thorax	1.96	0.8
Myosin 61F	Head	1.66	0.13
FBgn0010246	Thorax	29.34	3.52
Troponin C at 47D	Head	0.59	0.08
FBgn0010423	Thorax	4.49	0.81
Troponin C at 73F	Head	8.21	2.74
FBgn0010424	Thorax	32.13	13.12
Troponin C at 41C	Head	35.44	7.47
FBgn0013348	Thorax	170.08	37.33
Troponin C iso 4	Head	0.27	0.07
FBgn0033027	Thorax	191.36	49.53
Tropomyosin 1	Head	225.43	21.13
FBgn0003721	Thorax	544.97	26.69
Tropomyosin 2	Head	178.49	17.04
FBgn0004117	Thorax	456.79	42.14

Tissue-specific averaged RPKM values of select genes primarily expressed in muscle, neural and glial cells.

Table S3A. DAVID 976 genes with 123 Clusters

Neural Change Age	Term	Count	P value
Annotation Cluster 1 Enrichment Score: 3.98	UP_KEYWORDS Chaperone	11	0.002
	GOTERM_BP_DIRECT GO:0006457~protein folding	16	0.006
Annotation Cluster 2 Enrichment Score: 3.47	UP_KEYWORDS Transmembrane	242	0.0002
Annotation Cluster 3	Enrichment Score: 3.24 GOTERM_BP_DIRECT GO:0045087~innate immune response	19	0.0002
Annotation Cluster 4 Enrichment Score: 3.04	GOTERM_CC_DIRECT GO:0005576 extracellular region	53	0.0129
Annotation Cluster 5 Enrichment Score: 2.82	UP_KEYWORDS NAD	14	0.0001
Annotation Cluster 6 Enrichment Score: 2.79	KEGG_PATHWAY dme00071: Fatty acid degradation	12	0.00003
Annotation Cluster 7 Enrichment Score: 2.67	GOTERM_MF_DIRECT GO:0030170 pyridoxal phosphate binding	11	0.0001
Annotation Cluster 9 Enrichment Score: 2.35	KEGG_PATHWAY dme00280:Valine, leucine and isoleucine degradation	8	0.02
Annotation Cluster 10 Enrichment Score: 2.23	UP_KEYWORDS Mitochondrion	35	0.0001
Annotation Cluster 11 Enrichment Score: 2.23	COG_ONTOLOGY Secondary metabolites biosynthesis, transport, and catabolism	16	0.02
	GOTERM_CC_DIRECT GO:0005789 endoplasmic reticulum membrane	18	0.35
Annotation Cluster 12 Enrichment Score: 2.19	KEGG_PATHWAY dme00350: Tyrosine-Phenylalanine metabolism	8	0.0003
Annotation Cluster 13 Enrichment Score: 2.09	GOTERM_MF_DIRECT GO:0005524~ATP binding	68	0.04
Annotation Cluster 14 Enrichment Score: 2.04	INTERPRO IPR011009: Protein kinase-like domain	24	0.44
Annotation Cluster 15 Enrichment Score: 2.01	GOTERM_BP_DIRECT GO:0006096 glycolytic process	7	0.01
Annotation Cluster 16 Enrichment Score: 1.77	KEGG_PATHWAY dme00981:Insect hormone biosynthesis	5	0.04
Annotation Cluster 17 Enrichment Score: 1.69	GOTERM_CC_DIRECT GO:0031305 integral component of mitochondrial inner membrane	4	0.07
Annotation Cluster 18 Enrichment Score: 1.59	UP_SEQ_FEATURE signal peptide	45	0.00
	UP_KEYWORDS Glycoprotein	45	0.03
Annotation Cluster 19 Enrichment Score: 1.53	GOTERM_MF_DIRECT GO:0005328 neurotransmitter:sodium symporter activity	7	0.004
	GOTERM_MF_DIRECT GO:0005326 neurotransmitter transporter activity	4	0.15
Annotation Cluster 20 Enrichment Score: 1.51	PR001611:Leucine-rich repeat	14	0.08
Annotation Cluster 21 Enrichment Score: 1.47	IPR000873:AMP-dependent synthetase/ligase	6	0.05
Neural Change Age	Term	Count	PValue
Annotation Cluster 22	IPR013525:ABC-2 type transporter	6	0.00

Enrichment Score: 1.45			
Annotation Cluster 23 Enrichment Score: 1.45	GO:0050660~flavin adenine dinucleotide binding	11	0.01
Annotation Cluster 24 Enrichment Score: 1.44	GO:0007623~circadian rhythm	11	0.01
Annotation Cluster 25 Enrichment Score: 1.38	IPR013816: ATP-grasp fold, subdomain 2	5	0.01
Annotation Cluster 26 Enrichment Score: 1.37	IPR023210: NADP-dependent oxidoreductase domain	5	0.01
Annotation Cluster 27 Enrichment Score: 1.36	Collagen	3	0.02
Annotation Cluster 28 Enrichment Score: 1.33	GO:0007186~G-protein coupled receptor signaling pathway	11	0.51
Annotation Cluster 29 Enrichment Score: 1.31	UP_KEYWORDS Peroxidase	8	0.00
	GO:0045454~cell redox homeostasis	7	0.14
Annotation Cluster 30 Enrichment Score: 1.3	Electron transport	8	0.02

Table S3B. DAVID 1289 genes with 112 Clusters

Thorax Change Age	Term	Count	P value
Annotation Cluster 1 Enrichment Score: 4.12	UP_KEYWORDS Nucleotide-binding	104	2.20E-07
Annotation Cluster 2 Enrichment Score: 3.66	KEGG_PATHWAY dme00190: Oxidative phosphorylation	32	0.00
Annotation Cluster 3 Enrichment Score: 3.09	GOTERM_MF_DIRECT GO:0003735 Structural constituent of ribosome	43	0.06
Annotation Cluster 4 Enrichment Score: 2.85	UP_SEQ_FEATURE repeat:HEAT 6	6	3.96E-04
Annotation Cluster 5 Enrichment Score: 2.45	INTERPRO IPR009060:UBA-like	14	3.18E-05
Annotation Cluster 6 Enrichment Score: 2.35	GOTERM_MF_DIRECT GO:0005525~GTP binding	34	5.22E-04
Annotation Cluster 7 Enrichment Score: 2.18	INTERPRO IPR013816:ATP-grasp fold, subdomain 2	7	6.20E-04
Annotation Cluster 8 Enrichment Score: 2.07	INTERPRO IPR011993:Pleckstrin homology-like domain	33	5.06E-05
Annotation Cluster 9 Enrichment Score: 1.74	UP_KEYWORDS Kinase	40	0.002
Annotation Cluster 10 Enrichment Score: 1.66	GOTERM_MF_DIRECT GO:0003746 translation elongation factor activity	8	0.003
Annotation Cluster 11 Enrichment Score: 1.64	GOTERM_BP_DIRECT GO:0000902~cell morphogenesis	11	0.03
Annotation Cluster 12 Enrichment Score: 1.64	GOTERM_BP_DIRECT GO:0006511 ubiquitin-dependent protein catabolic process	14	0.02
Annotation Cluster 13 Enrichment Score: 1.59	UP_KEYWORDS Electron transport	8	0.09
Annotation Cluster 14 Enrichment Score: 1.52	UP_KEYWORDS Protein biosynthesis	19	4.24E-04
Annotation Cluster 15 Enrichment Score: 1.50	GOTERM_MF_DIRECT GO:0005096 GTPase activator activity	18	0.01
Annotation Cluster 16	UP_KEYWORDS Sensory transduction	16	0.31

Enrichment Score: 1.46			
Annotation Cluster 17 Enrichment Score: 1.45	GOTERM_BP_DIRECT GO:0007480 imaginal disc-derived leg morphogenesis	13	0.01
Annotation Cluster 18 Enrichment Score: 1.41	UP_KEYWORDS FAD	12	0.02
Annotation Cluster 19 Enrichment Score: 1.39	GOTERM_MF_DIRECT GO:0008601 Protein phosphatase type 2A regulator activity	5	0.01
Annotation Cluster 20 Enrichment Score: 1.39	GOTERM_BP_DIRECT GO:0008355 Olfactory learning	12	0.04
Annotation Cluster 21 Enrichment Score: 1.29	GOTERM_MF_DIRECT GO:0008017 Microtubule binding	19	0.02

Table S4. Wilcoxon P value Test of Transcriptional Drift Variance

	**Heads**			**Thorax**		
	**Total Genes**	**Age Response**	**IF Youthful**	**Total Genes**	**Age Response**	**IF Youthful**
**No. of Genes**	**9,800**	**1197**	**221**	**10,000**	**1,347**	**58**
**4W vs 1W**	**< 2.2E-16**	**< 2.2E-16**	**< 2.2E-16**	**< 2.2E-16**	**< 2.2E-16**	**0.12**
**4W-IF vs 1W**	**< 2.2E-16**	**< 2.2E-16**	**< 2.2E-16**	**< 2.2E-16**	**6.46E-06**	**0.15**
**4W-IF vs 4W**	**0.0015**	**< 2.2e-16**	**< 2.2E-16**	**0.75**	**0.0009**	**0.084**

Table S5. Head Transcriptome Neural Variance Changes (VC)

			VC			Z Scores		
**#**	**Gene Name**	**Gene ID**	**4W /4W-IF**	**4W /1W**	**4W-IF /1W**	**1W**	**4W**	**4W-IF**
**1**	***timeless***	**FBgn0014396**	**21.53**	**0.001**	**0.1**	**0.031**	**0.063**	**0.003**
**2**	***period***	**FBgn0003068**	**4.41**	**0.05**	**0.91**	**0.053**	**0.215**	**0.049**
**3**	***AP-2μ***	**FBgn0024832**	**5.85**	**0.01**	**0.83**	**0.017**	**0.085**	**0.015**
**4**	***Semaphorin 5c***	**FBgn0250876**	**4.92**	**0.05**	**1.25**	**0.042**	**0.258**	**0.052**
**5**	***Obp99a***	**FBgn0039678**	**3.27**	**0.43**	**1.57**	**0.271**	**1.392**	**0.426**
**6**	***CG14509***	**FBgn0039647**	**1.43**	**0.07**	**3.83**	**0.018**	**0.258**	**0.052**
**7**	***Innexin 2***	**FBgn0027108**	**11.82**	**0.02**	**0.36**	**0.059**	**0.251**	**0.021**
**8**	***neuralized***	**FBgn0002932**	**23.04**	**0.01**	**0.18**	**0.037**	**0.152**	**0.007**
**9**	***scab***	**FBgn0003328**	**4.94**	**0.06**	**1.14**	**0.05**	**0.281**	**0.057**
**10**	***moladietz***	**FBgn0086711**	**19.25**	**0.02**	**0.29**	**0.065**	**0.363**	**0.019**
**11**	***minibrain***	**FBgn0259168**	**8.33**	**0.01**	**1.76**	**0.008**	**0.111**	**0.013**
**12**	***Ebony***	**FBgn0000527**	**12.67**	**0.05**	**0.21**	**0.242**	**0.655**	**0.052**
**13**	***Fmr1***	**FBgn0028734**	**4.98**	**0.02**	**0.48**	**0.048**	**0.116**	**0.023**
**14**	***Sap 47kD***	**FBgn0013334**	**3.76**	**0.02**	**1.01**	**0.023**	**0.089**	**0.024**
**15**	***pale***	**FBgn0005626**	**3.62**	**0.18**	**2.38**	**0.076**	**0.653**	**0.181**
**16**	***shaggy***	**FBgn0003371**	**4.9**	**0.02**	**0.47**	**0.043**	**0.1**	**0.02**
**17**	***C kinase IIβ***	**FBgn0000259**	**4.33**	**0.02**	**2.24**	**0.007**	**0.066**	**0.015**
**18**	***NeuropeptideF***	**FBgn0027109**	**4.56**	**0.03**	**0.53**	**0.06**	**0.145**	**0.032**

Table S6. Thorax Transcriptome Lipid Metabolism Variance Changes (VC)

GO Term and Gene Name			VC			Z Scores		
#	Fatty acid metabolic procese	Gene ID	4W /1W	4W-IF /1W	4W /4W-IF	1W	4W	4W-IF
1	CG2781, fatty acid elongase 7	FBGN0037534	18.34	0.73	4.2	0.084	0.224	0.05
2	CG17544, acyl-Coenzyme A oxidase 3, pristanoyl	FBGN0032775	10.81	0.73	16.4	0.013	0.071	0.004
3	Fatty acid synthase 3	FBGN0040001	10.85	0.72	5.26	0.559	0.351	0.067
4	stearoyl-CoA desaturase	FBGN0039755	33.72	0.72	3.97	0.817	0.498	0.125
5	acyl-CoA synthetase medium-chain family member 3	FBGN0039156	21.33	0.73	9.08	0.133	0.122	0.014
6	fatty acid elongase	FBGN0053110	16.4	0.73	6.04	0.384	0.202	0.034
	Glycerophospholipid met							
7	Dihydroxyacetone phosphate acyltransferase	FBGN0040212	6.46	0.73	89.72	0.132	0.093	0.002
8	phospholipase A2 group XV	FBGN0051683	4.14	0.73	5.23	0.691	0.064	0.012
9	qless, coenzyme Q1	FBGN0051005	8.11	0.73	20.86	0.042	0.127	0.006
10	dolichol kinase	FBGN0034141	18.53	0.73	7.6	0.135	0.057	0.008
11	Phosphatidylinositol synthase	FBGN0030670	43.02	0.73	8.03	0.105	0.054	0.007
12	ELOVL fatty acid elongase 7	FBGN0039030	6.56	0.72	6.5	0.507	0.531	0.082
13	phosphatidylglycerophosphate synthase 1	FBGN0038649	5.01	0.73	17.15	0.001	0.044	0.003
14	phosphatidylglycerophosphate synthase 1	FBGN0034971	37.34	0.73	3.92	0.003	0.058	0.015
	Glycerolipid metabolism							
15	Lipase family member N	FBGN0038070	0.09	0.45	11.57	1.045	0.566	0.054
16	fu12, 1-acylglycerol-3-phosphate O-acyltransferase 2	FBGN0026718	6.47	0.73	7.11	0.092	0.122	0.018
17	aldo-keto reductase family 1 member A1	FBGN0037537	28.56	0.73	10.9	0.106	0.131	0.012
18	1-acylglycerol-3-phosphate O-acyltransferase 4	FBGN0036622	13.2	0.73	9.29	0.28	0.047	0.005

Table S7A. Proteolytic Genes

#	Gene ID	Name	Tissue	4W/ 4W-IF		4W / 1W		4W-IF / 1W	
A	Proteasome			VC	FC	VC	FC	VC	FC
1	FBgn0015282	*Rpt2*	*H	4.22	1.11	10.75	1.31	2.55	1.18
			**T	0.20	1.09	0.12	1.52	0.58	1.39
2	FBgn0250746	*Prosβ7*	H	7.23	1.15	1.61	1.36	0.22	1.19
			T	1.11	1.35	1.95	2.47	1.75	1.83
3	FBgn0028695	*Rpn1*	H	16.15	1.13	9.08	1.38	0.56	1.22
			T	1.79	1.21	2.04	1.93	1.14	1.60
4	FBgn0028690	*Rpn5*	H	5.14	1.14	3.95	1.37	0.77	1.20
			T	1.21	1.25	0.21	1.66	0.18	1.33
5	FBgn0028689	*Rpn6*	H	7.32	1.11	4.62	1.27	0.63	1.14
			T	2.72	1.20	0.86	1.66	0.32	1.38
6	FBgn0028688	*Rpn7*	H	4.10	1.03	5.65	1.21	1.38	1.18
			T	1.03	1.18	0.90	1.38	0.88	1.17
7	FBgn0015283	*Rpn10*	H	6.03	1.16	7.25	1.46	1.20	1.26
			T	1.00	1.28	0.53	1.94	0.53	1.51
8	FBgn0020369	*Rpt6*	H	16.39	1.13	2.08	1.41	0.13	1.25
			T	3.24	1.27	1.23	2.28	0.38	1.80
9	FBgn0004066	*Prosα4*	H	5.11	1.14	2.14	1.48	0.42	1.30
			T	1.73	1.32	1.42	2.37	0.82	1.79
10	FBgn0016697	*Prosα5*	H	3.80	1.16	1.40	1.30	0.37	1.12
			T	1.50	1.45	1.99	2.86	1.33	1.98
11	FBgn0028691	*Rpn9*	H	9.89	1.19	11.10	1.36	1.12	1.14
			T	0.66	1.34	1.61	1.96	2.43	1.46
12	FBgn0028694	*Rpn11*	H	7.21	1.18	3.76	1.45	0.52	1.22
			T	3.14	1.29	2.73	1.76	0.87	1.37
13	FBgn0028685	*Rpt4*	H	14.06	1.13	5.80	1.36	0.41	1.20
			T	0.29	1.22	0.09	1.83	0.32	1.50
14	FBgn0028684	*Rpt5*	H	3.83	1.11	15.99	1.31	4.18	1.19
			T	5.39	1.27	0.33	1.89	0.06	1.49
15	FBgn0028687	*Rpt1*	H	4.00	1.06	3.91	1.30	0.98	1.23
			T	2.80	1.29	2.11	2.10	0.75	1.63
16	FBgn0028686	*Rpt3*	H	7.08	1.10	5.05	1.49	0.71	1.35
			T	4.70	1.45	0.52	2.91	0.11	2.01
17	FBgn0028692	*Rpn2*	H	5.97	1.14	12.33	1.30	2.06	1.14
			T	0.19	1.22	0.25	2.05	1.36	1.68
18	FBgn0261396	*Rpn3*	H	24.59	1.14	5.24	1.33	0.21	1.17
			T	0.86	1.22	1.09	2.33	1.26	1.91
19	FBgn0036994	CG5199	H	6.22	1.22	1.00	1.71	0.16	1.40
			T	0.87	1.21	1.01	1.62	1.15	1.34

Table S7B. Lysosomal Genes

#	Gene ID	Name	Tissue	4W/ 4W-IF		4W / 1W		4W-IF / 1W	
B	Lysosome			VC	FC	VC	FC	VC	FC
20	FBgn0010435	*Emp*	H	5.37	0.83	4.11	0.59	0.77	0.71
			T	0.68	0.96	0.49	0.76	0.73	0.79
21	FBgn0013770	*Cpl,CathL*	H	10.12	0.91	41.36	1.26	4.09	1.39
			T	0.79	1.18	0.27	1.19	0.34	1.01
22	FBgn0000416	*Sap-r*	H	6.06	0.87	1.89	0.93	0.31	1.07
			T	0.56	0.95	0.44	1.09	0.78	1.15
23	FBgn0030141	*Gga*	H	6.81	1.04	0.91	1.28	0.13	1.24
			T	0.05	0.97	0.00	1.36	0.07	1.40
24	FBgn0051072	*Lerp*	H	5.29	1.03	2.47	1.24	0.47	1.21
			T	0.80	1.02	0.26	1.05	0.33	1.03
25	FBgn0030452	*MFS10*	H	3.77	0.92	0.82	0.98	0.22	1.07
			T	6.36	1.03	1.32	1.20	0.21	1.17
26	FBgn0000319	*Chc*	H	4.96	0.92	3.18	0.96	0.64	1.05
			T	2.08	1.01	4.68	1.29	2.25	1.28
27	FBgn0013750	*Arf51F*	H	4.75	0.94	2.65	0.91	0.56	0.96
			T	1.23	1.22	0.07	1.28	0.05	1.05
28	FBgn0086656	*shrb, Vps32*	H	3.86	1.11	0.83	1.42	0.22	1.29
			T	3.81	1.21	1.63	1.59	0.43	1.32
29	FBgn0026479	*Drp1*	H	4.33	1.02	1.92	1.13	0.44	1.11
			T	0.95	1.06	1.35	1.43	1.42	1.35
30	FBgn0016693	*Past1*	H	3.76	0.91	7.60	0.96	2.02	1.05
			T	0.16	1.12	0.46	1.38	2.80	1.23
31	FBgn0016038	*Vsp37A*	H	5.01	1.04	5.87	1.25	1.17	1.20
			T	0.14	1.05	0.90	1.39	6.23	1.32
32	FBgn0034443	*cer*	H	5.03	0.84	0.72	1.09	0.14	1.30
			T	1.32	1.09	0.72	1.12	0.54	1.03
33	FBgn0031563	*CG10031*	H	4.13	1.03	1.31	1.14	0.32	1.10
			T	0.37	1.14	1.00	1.28	2.68	1.12
34	FBgn0031897	*CG13784*	H	4.96	0.83	1.78	0.73	0.36	0.88
			T	1.63	0.74	7.24	0.74	4.45	0.99
35	FBgn0039054	*cow*	H	8.42	1.00	1.08	1.00	0.13	1.01
			T	0.27	0.83	0.18	0.69	0.70	0.83
36	FBgn0250848	*26-29-p*	H	4.61	0.82	1.60	0.80	0.35	0.98
			T	2.18	0.95	3.97	0.98	1.82	1.03
37	FBgn0243511	*Psidin*	H	5.08	1.00	1.65	1.25	0.33	1.24
			T	2.94	0.85	1.95	1.24	0.66	1.46

Table S7C. Autophagy Genes

#	Gene ID	Name	Tissue	4W/ 4W-IF		4W / 1W		4W-IF / 1W	
C	Autophagy			VC	FC	VC	FC	VC	FC
38	FBgn0015277	*Pi3K59F*	H	4.41	1.01	2.58	1.47	0.59	1.45
			T	0.30	0.96	0.27	1.47	0.90	1.53
39	FBgn0026479	*Drp1*	H	4.33	1.02	1.92	1.13	0.44	1.11
			T	0.95	1.06	1.35	1.43	1.42	1.35
40	FBgn0000567	*Eip74EF*	H	5.00	1.13	4.88	1.13	0.98	1.00
			T	0.27	1.03	0.23	1.45	0.83	1.41

41	FBgn0010638	*Sec61β*	H	4.25	0.99	2.49	1.16	0.59	1.17
			T	1.69	1.06	0.17	1.37	0.10	1.29
42	FBgn0034110	*Atg9*	H	4.61	1.05	0.99	1.02	0.22	0.98
			T	0.52	0.89	0.24	0.89	0.46	1.00
43	FBgn0039705	*Atg16L2*	H	6.91	0.83	1.19	1.09	0.17	1.31
			T	1.17	0.99	0.37	1.18	0.32	1.19
44	FBgn0040780	*Atg10*	H	10.97	1.13	4.13	1.00	0.38	0.88
			T	0.05	0.94	0.08	0.88	1.67	0.94

Table S7D. Ubiquitination Genes

#	Gene ID	Name	Tissue	4W/ 4W-IF		4W / 1W		4W-IF / 1W	
D	Ubiquitin			VC	FC	VC	FC	VC	FC
45	FBgn0027052	*STUB1*	H	4.72	1.14	0.97	1.20	0.20	1.05
			T	2.93	1.29	1.78	1.47	0.61	1.15
46	FBgn0261268	*Cullin3*	H	3.99	0.99	0.97	1.05	0.24	1.06
			T	1.56	1.03	0.60	1.18	0.38	1.15
47	FBgn0041174	*VHL*	H	5.77	1.06	1.89	1.40	0.33	1.32
			T	0.63	1.11	0.65	1.58	1.03	1.43
48	FBgn0039875	*Synoviolin1*	H	4.96	0.92	3.31	0.97	0.67	1.05
			T	1.39	0.91	1.02	1.13	0.74	1.24
49	FBgn0028467	*UBE4A*	H	4.18	1.03	0.91	1.07	0.22	1.04
			T	3.25	0.93	1.40	1.07	0.43	1.14
50	FBgn0032467	*UBE4B*	H	27.68	1.43	6.93	1.61	0.25	1.13
			T	8.76	1.19	0.13	1.03	0.01	0.86
51	FBgn0030863	*UBE2S*	H	12.77	1.04	0.95	1.17	0.07	1.13
			T	0.23	1.17	0.21	1.46	0.91	1.25

Table S8. Head and Thorax VZ Scores for Proteolytic Genes

		Head			Thorax		
		Corrected VZ Scores			Corrected VZ Scores		
Gene #	A. Proteasome	1W	4W	4W-IF	1W	4W	4W-IF
1	*Rpt2*	0.018	0.193	0.046	0.052	0.006	0.03
2	*Prosβ7*	0.061	0.098	0.014	0.045	0.087	0.078
3	*Rpn1*	0.037	0.333	0.021	0.012	0.024	0.014
4	*Rpn5*	0.042	0.166	0.032	0.108	0.023	0.019
5	*Rpn6*	0.033	0.155	0.021	0.054	0.047	0.017
6	*Rpn7*	0.041	0.231	0.056	0.064	0.058	0.056
7	*Rpn10*	0.024	0.177	0.029	0.031	0.016	0.016
8	*Rpt6*	0.073	0.152	0.009	0.069	0.085	0.026
9	*Prosα4*	0.069	0.147	0.029	0.076	0.108	0.063
10	*Prosα5*	0.11	0.154	0.04	0.07	0.14	0.093
11	*Rpn9*	0.026	0.29	0.029	0.031	0.051	0.076
12	*Rpn11*	0.059	0.221	0.031	0.021	0.058	0.018
13	*Rpt4*	0.042	0.244	0.017	0.114	0.011	0.037
14	*Rpt5*	0.012	0.194	0.051	0.053	0.018	0.003
15	*Rpt1*	0.029	0.112	0.028	0.039	0.082	0.029
16	*Rpt3*	0.05	0.251	0.035	0.106	0.055	0.012
17	*Rpn2*	0.021	0.257	0.043	0.028	0.007	0.038
18	*Rpn3*	0.044	0.229	0.009	0.065	0.071	0.082
	**B. Lysosome**						
19	*CG5199*	0.144	0.144	0.023	0.092	0.093	0.106
20	*emp*	0.043	0.178	0.033	0.126	0.062	0.092
21	*Cp1, Cath L*	0.003	0.129	0.013	0.346	0.093	0.117
22	*Sap-r*	0.097	0.184	0.03	0.157	0.069	0.123
23	*Gga*	0.062	0.057	0.008	0.153	0.001	0.011
24	*Lerp*	0.026	0.065	0.012	0.164	0.043	0.054
25	*MFS10*	0.076	0.062	0.016	0.051	0.068	0.011
26	*Chc*	0.04	0.127	0.026	0.02	0.091	0.044
27	*Arf51F*	0.051	0.135	0.028	0.091	0.006	0.005
28	*Vps32, ESCRT-III*	0.114	0.095	0.025	0.039	0.064	0.017
29	*Drp1*	0.042	0.08	0.018	0.032	0.043	0.045
30	*Past1*	0.023	0.177	0.047	0.036	0.017	0.101
31	*Vsp37A*	0.023	0.134	0.027	0.016	0.014	0.099
32	*cer*	0.079	0.057	0.011	0.143	0.103	0.078
33	*CG10031*	0.119	0.156	0.038	0.084	0.084	0.224
34	*CG13784*	0.057	0.101	0.02	0.01	0.07	0.043
35	*Cow*	0.118	0.127	0.015	0.1	0.019	0.07
36	*26-29-p*	0.083	0.133	0.029	0.032	0.129	0.059
37	*psidin*	0.042	0.069	0.014	0.067	0.13	0.044
	**C. Autophagy**						
38	*Pi3K59F*	0.032	0.084	0.019	0.047	0.013	0.042
39	*Drp1*	0.042	0.08	0.018	0.032	0.043	0.045
40	*Eip74EF*	0.047	0.231	0.046	0.053	0.012	0.044
41	*Sec61β*	0.022	0.056	0.013	0.113	0.019	0.011
42	*Atg9*	0.069	0.068	0.015	0.154	0.037	0.071
43	*ATG16L2*	0.09	0.107	0.015	0.055	0.02	0.017
44	*Atg10*	0.053	0.22	0.02	0.06	0.005	0.1

	**D. Ubiquitin**						
**45**	***STUB1***	0.083	0.08	0.017	0.014	0.025	0.008
**46**	***Cullin 3***	0.062	0.06	0.015	0.12	0.071	0.046
**47**	***VHL***	0.06	0.113	0.02	0.044	0.029	0.045
**48**	***Synoviolin1***	0.029	0.094	0.019	0.08	0.082	0.059
**49**	***UBE4A***	0.045	0.041	0.01	0.085	0.119	0.037
**50**	***UBE4B***	0.057	0.392	0.014	0.319	0.041	0.005
**51**	***UBE2S***	0.046	0.044	0.003	0.088	0.018	0.08

References

1. Ratliff EP, Kotzebue RW, Molina B, Mauntz RE, Gonzalez A, Barekat A, El-Mecharrafie N, Garza S, Gurney MA, Achal M, Linton PJ, Harris GL and Finley KD (2016) Assessing Basal and Acute Autophagic Responses in the Adult Drosophila Nervous System: The Impact of Gender, Genetics and Diet on Endogenous Pathway Profiles. PLoS One 11:e0164239. doi: 10.1371/journal.pone.0164239
2. Ratliff EP, Mauntz RE, Kotzebue RW, Gonzalez A, Achal M, Barekat A, Finley KA, Sparhawk JM, Robinson JE, Herr DR, Harris GL, Joiner WJ and Finley KD (2015) Aging and Autophagic Function Influences the Progressive Decline of Adult Drosophila Behaviors. PLoS One 10:e0132768. doi: 10.1371/journal.pone.0132768
3. Barekat A, Gonzalez A, Mauntz RE, Kotzebue RW, Molina B, El-Mecharrafie N, Conner CJ, Garza S, Melkani GC, Joiner WJ, Lipinski MM, Finley KD and Ratliff EP (2016) Using Drosophila as an integrated model to study mild repetitive traumatic brain injury. Sci Rep 6:25252. doi: 10.1038/srep25252
4. Simonsen A, Cumming RC, Brech A, Isakson P, Schubert DR and Finley KD (2008) Promoting basal levels of autophagy in the nervous system enhances longevity and oxidant resistance in adult Drosophila. Autophagy 4:176-84. doi: 5269 [pii]
5. Bartlett BJ, Isakson P, Lewerenz J, Sanchez H, Kotzebue RW, Cumming RC, Harris GL, Nezis IP, Schubert DR, Simonsen A and Finley KD (2011) p62, Ref(2)P and ubiquitinated proteins are conserved markers of neuronal aging, aggregate formation and progressive autophagic defects. Autophagy 7. doi: 14943 [pii]
6. Currais A, Fischer W, Maher P and Schubert D (2017) Intraneuronal protein aggregation as a trigger for inflammation and neurodegeneration in the aging brain. FASEB J 31:5-10. doi: 10.1096/fj.201601184
7. Goldberg J, Currais A, Prior M, Fischer W, Chiruta C, Ratliff E, Daugherty D, Dargusch R, Finley K, Esparza-Molto PB, Cuezva JM, Maher P, Petrascheck M and Schubert D (2018) The mitochondrial ATP synthase is a shared drug target for aging and dementia. Aging Cell. doi: 10.1111/acel.12715
8. Tariq MA, Kim HJ, Jejelowo O and Pourmand N (2011) Whole-transcriptome RNAseq analysis from minute amount of total RNA. Nucleic Acids Res 39:e120. doi: 10.1093/nar/gkr547
9. Schafer MJ, Dolgalev I, Alldred MJ, Heguy A and Ginsberg SD (2015) Calorie Restriction Suppresses Age-Dependent Hippocampal Transcriptional Signatures. PLoS One 10:e0133923. doi: 10.1371/journal.pone.0133923
10. Gill S, Le HD, Melkani GC and Panda S (2015) Time-restricted feeding attenuates age-related cardiac decline in Drosophila. Science 347:1265-9. doi: 10.1126/science.1256682
11. Landis GN, Salomon MP, Keroles D, Brookes N, Sekimura T and Tower J (2015) The progesterone antagonist mifepristone/RU486 blocks the negative effect on life span caused by mating in female Drosophila. Aging (Albany NY) 7:53-69. doi: 10.18632/aging.100721
12. Ramskold D, Wang ET, Burge CB and Sandberg R (2009) An abundance of ubiquitously expressed genes revealed by tissue transcriptome sequence data. PLoS Comput Biol 5:e1000598. doi: 10.1371/journal.pcbi.1000598
13. Labaj PP and Kreil DP (2016) Sensitivity, specificity, and reproducibility of RNA-Seq differential expression calls. Biol Direct 11:66. doi: 10.1186/s13062-016-0169-7
14. Lerch JK, Kuo F, Motti D, Morris R, Bixby JL and Lemmon VP (2012) Isoform diversity and regulation in peripheral and central neurons revealed through RNA-Seq. PLoS One 7:e30417. doi: 10.1371/journal.pone.0030417
15. Trapnell C, Williams BA, Pertea G, Mortazavi A, Kwan G, van Baren MJ, Salzberg SL, Wold BJ and Pachter L (2010) Transcript assembly and quantification by RNA-Seq reveals unannotated transcripts and isoform switching during cell differentiation. Nat Biotechnol 28:511-5. doi: 10.1038/nbt.1621

16. Wang L, Karpac J and Jasper H (2014) Promoting longevity by maintaining metabolic and proliferative homeostasis. J Exp Biol 217:109-18. doi: 10.1242/jeb.089920
17. Rangaraju S, Solis GM, Thompson RC, Gomez-Amaro RL, Kurian L, Encalada SE, Niculescu AB, 3rd, Salomon DR and Petrascheck M (2015) Suppression of transcriptional drift extends C. elegans lifespan by postponing the onset of mortality. Elife 4:e08833. doi: 10.7554/eLife.08833
18. Currais A, Goldberg J, Farrokhi C, Chang M, Prior M, Dargusch R, Daugherty D, Armando A, Quehenberger O, Maher P and Schubert D (2015) A comprehensive multiomics approach toward understanding the relationship between aging and dementia. Aging (Albany NY) 7:937-55. doi: 10.18632/aging.100838
19. Wang L, Ryoo HD, Qi Y and Jasper H (2015) PERK Limits Drosophila Lifespan by Promoting Intestinal Stem Cell Proliferation in Response to ER Stress. PLoS Genet 11:e1005220. doi: 10.1371/journal.pgen.1005220
20. Drosophila 12 Genomes C, Clark AG, Eisen MB, Smith DR, Bergman CM, Oliver B, Markow TA, Kaufman TC, Kellis M, Gelbart W, Iyer VN, Pollard DA, Sackton TB, Larracuente AM, Singh ND, Abad JP, Abt DN, Adryan B, Aguade M, Akashi H, Anderson WW, Aquadro CF, Ardell DH, Arguello R, Artieri CG, Barbash DA, Barker D, Barsanti P, Batterham P, Batzoglou S, Begun D, Bhutkar A, Blanco E, Bosak SA, Bradley RK, Brand AD, Brent MR, Brooks AN, Brown RH, Butlin RK, Caggese C, Calvi BR, Bernardo de Carvalho A, Caspi A, Castrezana S, Celniker SE, Chang JL, Chapple C, Chatterji S, Chinwalla A, Civetta A, Clifton SW, Comeron JM, Costello JC, Coyne JA, Daub J, David RG, Delcher AL, Delehaunty K, Do CB, Ebling H, Edwards K, Eickbush T, Evans JD, Filipski A, Findeiss S, Freyhult E, Fulton L, Fulton R, Garcia AC, Gardiner A, Garfield DA, Garvin BE, Gibson G, Gilbert D, Gnerre S, Godfrey J, Good R, Gotea V, Gravely B, Greenberg AJ, Griffiths-Jones S, Gross S, Guigo R, Gustafson EA, Haerty W, Hahn MW, Halligan DL, Halpern AL, Halter GM, Han MV, Heger A, Hillier L, Hinrichs AS, Holmes I, Hoskins RA, Hubisz MJ, Hultmark D, Huntley MA, Jaffe DB, Jagadeeshan S, Jeck WR, Johnson J, Jones CD, Jordan WC, Karpen GH, Kataoka E, Keightley PD, Kheradpour P, Kirkness EF, Koerich LB, Kristiansen K, Kudrna D, Kulathinal RJ, Kumar S, Kwok R, Lander E, Langley CH, Lapoint R, Lazzaro BP, Lee SJ, Levesque L, Li R, Lin CF, Lin MF, Lindblad-Toh K, Llopart A, Long M, Low L, Lozovsky E, Lu J, Luo M, Machado CA, Makalowski W, Marzo M, Matsuda M, Matzkin L, McAllister B, McBride CS, McKernan B, McKernan K, Mendez-Lago M, Minx P, Mollenhauer MU, Montooth K, Mount SM, Mu X, Myers E, Negre B, Newfeld S, Nielsen R, Noor MA, O'Grady P, Pachter L, Papaceit M, Parisi MJ, Parisi M, Parts L, Pedersen JS, Pesole G, Phillippy AM, Ponting CP, Pop M, Porcelli D, Powell JR, Prohaska S, Pruitt K, Puig M, Quesneville H, Ram KR, Rand D, Rasmussen MD, Reed LK, Reenan R, Reily A, Remington KA, Rieger TT, Ritchie MG, Robin C, Rogers YH, Rohde C, Rozas J, Rubenfield MJ, Ruiz A, Russo S, Salzberg SL, Sanchez-Gracia A, Saranga DJ, Sato H, Schaeffer SW, Schatz MC, Schlenke T, Schwartz R, Segarra C, Singh RS, Sirot L, Sirota M, Sisneros NB, Smith CD, Smith TF, Spieth J, Stage DE, Stark A, Stephan W, Strausberg RL, Strempel S, Sturgill D, Sutton G, Sutton GG, Tao W, Teichmann S, Tobari YN, Tomimura Y, Tsolas JM, Valente VL, Venter E, Venter JC, Vicario S, Vieira FG, Vilella AJ, Villasante A, Walenz B, Wang J, Wasserman M, Watts T, Wilson D, Wilson RK, Wing RA, Wolfner MF, Wong A, Wong GK, Wu CI, Wu G, Yamamoto D, Yang HP, Yang SP, Yorke JA, Yoshida K, Zdobnov E, Zhang P, Zhang Y, Zimin AV, Baldwin J, Abdouelleil A, Abdulkadir J, Abebe A, Abera B, Abreu J, Acer SC, Aftuck L, Alexander A, An P, Anderson E, Anderson S, Arachi H, Azer M, Bachantsang P, Barry A, Bayul T, Berlin A, Bessette D, Bloom T, Blye J, Boguslavskiy L, Bonnet C, Boukhgalter B, Bourzgui I, Brown A, Cahill P, Channer S, Cheshatsang Y, Chuda L, Citroen M, Collymore A, Cooke P, Costello M, D'Aco K, Daza R, De Haan G, DeGray S, DeMaso C, Dhargay N, Dooley K, Dooley E, Doricent M, Dorje P, Dorjee K, Dupes A, Elong R, Falk J, Farina A, Faro S, Ferguson D, Fisher S, Foley CD, Franke A, Friedrich D, Gadbois L, Gearin G, Gearin CR, Giannoukos G, Goode T, Graham J, Grandbois E, Grewal S, Gyaltsen K, Hafez N, Hagos B, Hall J, Henson C, Hollinger A, Honan T, Huard MD, Hughes L, Hurhula B, Husby ME, Kamat A, Kanga B, Kashin S, Khazanovich D, Kisner P, Lance K, Lara M, Lee W, Lennon N, Letendre F, LeVine R, Lipovsky A, Liu X, Liu J, Liu S, Lokyitsang T, Lokyitsang Y, Lubonja R, Lui A, MacDonald P, Magnisalis V, Maru K, Matthews C, McCusker W, McDonough S, Mehta T, Meldrim J, Meneus L, Mihai O, Mihalev A, Mihova T, Mittelman R, Mlenga V, Montmayeur A, Mulrain L, Navidi A, Naylor J, Negash T, Nguyen T, Nguyen N, Nicol R, Norbu C, Norbu N, Novod N, O'Neill B, Osman S, Markiewicz E, Oyono OL, Patti C, Phunkhang P, Pierre F, Priest M, Raghuraman S, Rege F, Reyes R, Rise C, Rogov P, Ross K, Ryan E, Settipalli S, Shea T, Sherpa N, Shi L, Shih D, Sparrow T, Spaulding J, Stalker J, Stange-Thomann N, Stavropoulos S, Stone C, Strader C, Tesfaye S,

Thomson T, Thoulutsang Y, Thoulutsang D, Topham K, Topping I, Tsamla T, Vassiliev H, Vo A, Wangchuk T, Wangdi T, Weiand M, Wilkinson J, Wilson A, Yadav S, Young G, Yu Q, Zembek L, Zhong D, Zimmer A, Zwirko Z, Jaffe DB, Alvarez P, Brockman W, Butler J, Chin C, Gnerre S, Grabherr M, Kleber M, Mauceli E and MacCallum I (2007) Evolution of genes and genomes on the Drosophila phylogeny. Nature 450:203-18. doi: 10.1038/nature06341

21. Haelterman NA, Jiang L, Li Y, Bayat V, Sandoval H, Ugur B, Tan KL, Zhang K, Bei D, Xiong B, Charng WL, Busby T, Jawaid A, David G, Jaiswal M, Venken KJ, Yamamoto S, Chen R and Bellen HJ (2014) Large-scale identification of chemically induced mutations in Drosophila melanogaster. Genome Res 24:1707-18. doi: 10.1101/gr.174615.114
22. Yamamoto S, Jaiswal M, Charng WL, Gambin T, Karaca E, Mirzaa G, Wiszniewski W, Sandoval H, Haelterman NA, Xiong B, Zhang K, Bayat V, David G, Li T, Chen K, Gala U, Harel T, Pehlivan D, Penney S, Vissers LE, de Ligt J, Jhangiani SN, Xie Y, Tsang SH, Parman Y, Sivaci M, Battaloglu E, Muzny D, Wan YW, Liu Z, Lin-Moore AT, Clark RD, Curry CJ, Link N, Schulze KL, Boerwinkle E, Dobyns WB, Allikmets R, Gibbs RA, Chen R, Lupski JR, Wangler MF and Bellen HJ (2014) A drosophila genetic resource of mutants to study mechanisms underlying human genetic diseases. Cell 159:200-14. doi: 10.1016/j.cell.2014.09.002
23. Ivanisevic J, Stauch KL, Petrascheck M, Benton HP, Epstein AA, Fang M, Gorantla S, Tran M, Hoang L, Kurczy ME, Boska MD, Gendelman HE, Fox HS and Siuzdak G (2016) Metabolic drift in the aging brain. Aging (Albany NY) 8:1000-20. doi: 10.18632/aging.100961
24. Simonsen A, Cumming RC and Finley KD (2007) Linking lysosomal trafficking defects with changes in aging and stress response in Drosophila. Autophagy 3:499-501. doi: 4604 [pii]
25. Walls SM, Jr., Attle SJ, Brulte GB, Walls ML, Finley KD, Chatfield DA, Herr DR and Harris GL (2013) Identification of sphingolipid metabolites that induce obesity via misregulation of appetite, caloric intake and fat storage in Drosophila. PLoS Genet 9:e1003970. doi: 10.1371/journal.pgen.1003970
26. Arrese EL, Patel RT and Soulages JL (2006) The main triglyceride-lipase from the insect fat body is an active phospholipase A(1): identification and characterization. J Lipid Res 47:2656-67. doi: 10.1194/jlr.M600161-JLR200
27. Xu K, Zheng X and Sehgal A (2008) Regulation of feeding and metabolism by neuronal and peripheral clocks in Drosophila. Cell Metab 8:289-300. doi: 10.1016/j.cmet.2008.09.006
28. Zirin J, Nieuwenhuis J and Perrimon N (2013) Role of autophagy in glycogen breakdown and its relevance to chloroquine myopathy. PLoS Biol 11:e1001708. doi: 10.1371/journal.pbio.1001708
